# ALDH1A3-acetaldehyde metabolism potentiates transcriptional heterogeneity in melanoma

**DOI:** 10.1016/j.celrep.2024.114406

**Published:** 2024-07-03

**Authors:** Yuting Lu, Jana Travnickova, Mihaly Badonyi, Florian Rambow, Andrea Coates, Zaid Khan, Jair Marques, Laura C. Murphy, Pablo Garcia-Martinez, Richard Marais, Pakavarin Louphrasitthiphol, Alex H.Y. Chan, Christopher J. Schofield, Alex von Kriegsheim, Joseph A. Marsh, Valeria Pavet, Owen J. Sansom, Robert S. Illingworth, E. Elizabeth Patton

**Affiliations:** 1 MRC Human Genetics Unit, Institute of Genetics and Cancer, The University of Edinburgh, Edinburgh EH4 2XU, UK; 2 Edinburgh Cancer Research, CRUK Scotland Centre, Institute of Genetics and Cancer, The University of Edinburgh, Edinburgh EH4 2XR, UK; 3 Department of Applied Computational Cancer Research, Institute for AI in Medicine (IKIM), University Hospital Essen, 45131 Essen, Germany; 4 University of Duisburg-Essen, 45141 Essen, Germany; 5 Insitute of Genetics and Cancer, The Univeristy of Edinburgh, Edinburgh, EH4 2XU, UK; 6 Cancer Research UK Manchester Institute, The University of Manchester, Alderley Park SK10 4TG, UK; 7 Oncodrug Ltd, Alderley Park, Macclesfield SK10 4TG, UK; 8 Ludwig Institute for Cancer Research, Nuffield Department of Clinical Medicine, University of Oxford, Headington, Oxford OX3 7DQ, UK; 9 Cancer Research UK Beatson Institute, CRUK Scotland Centre, Garscube Estate, Switchback Road, Bearsden Glasgow G61 1BD, UK; 10 School of Cancer Sciences, University of Glasgow, Glasgow G12 0ZD, UK; 11 Centre for Regenerative Medicine, Institute for Regeneration and Repair, The University of Edinburgh, Edinburgh BioQuarter, Edinburgh EH16 4UU, UK; 12 Department of Chemistry and the Ineos Oxford Institute for Antimicrobial Research, Chemistry Research Laboratory, University of Oxford, 12 Mansfield Road, Oxford OX1 5JJ, UK

**Keywords:** melanoma, pyruvate metabolism, acetaldehyde, ALDH1A3, ACSS2, TFAP2B, neural crest stem cell, residual disease, Nifuroxazide

## Abstract

Cancer cellular heterogeneity and therapy resistance arise substantially from metabolic and transcriptional adaptations, but how these are interconnected is poorly understood. Here, we show that, in melanoma, the cancer stem cell marker aldehyde dehydrogenase 1A3 (ALDH1A3) forms an enzymatic partnership with acetyl-coenzyme A (CoA) synthetase 2 (ACSS2) in the nucleus to couple high glucose metabolic flux with acetyl-histone H3 modification of neural crest (NC) lineage and glucose metabolism genes. Importantly, we show that acetaldehyde is a metabolite source for acetyl-histone H3 modification in an ALDH1A3-dependent manner, providing a physiologic function for this highly volatile and toxic metabolite. In a zebrafish melanoma residual disease model, an ALDH1-high subpopulation emerges following BRAF inhibitor treatment, and targeting these with an ALDH1 suicide inhibitor, nifuroxazide, delays or prevents BRAF inhibitor drug-resistant relapse. Our work reveals that the ALDH1A3-ACSS2 couple directly coordinates nuclear acetaldehyde-acetyl-CoA metabolism with specific chromatin-based gene regulation and represents a potential therapeutic vulnerability in melanoma.

## Highlights

• ALDH-high metabolic activity controls stem cell transcriptional states
• Nuclear ALDH1A3 partners with ACSS2 to promote selective acetyl-histone H3
• Acetaldehyde is an acetyl source for ALDH1A3-dependent histone H3 acetylation
• ALDH1A3 is a master regulator and drug target of melanoma heterogeneity

## Graphical abstract

**Figure:**
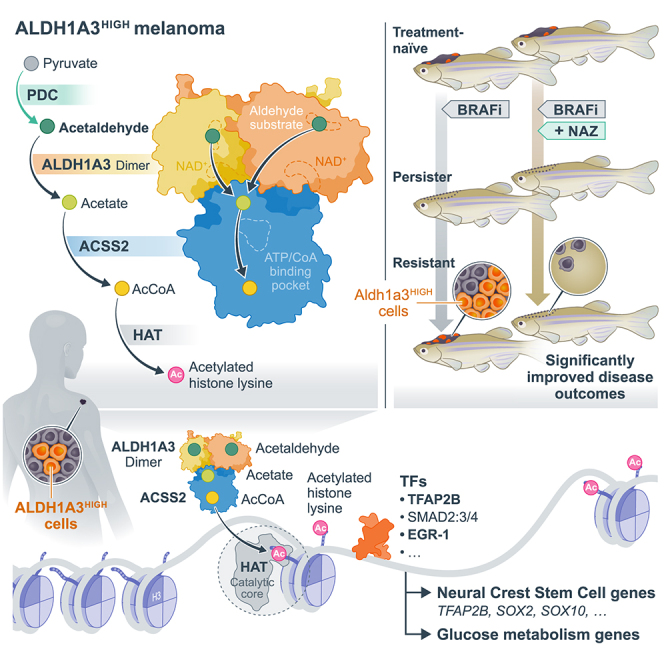

## Teaser

Metabolic and transcriptional cellular heterogeneity drives melanoma drug resistance and progression. This study reveals that ALDH1A3-ACSS2 directly coordinates nuclear acetaldehyde-acetyl-CoA metabolism with histone H3 acetylation and is associated with *TFAP2B*-neural crest stem cell and glucose metabolism gene expression. The ALDH1A3 metabolism-stem cell axis represents a potential therapeutic vulnerability in melanoma.

## Introduction

The perennial challenge in cancer therapies is that non-genetic mechanisms can potentiate dynamic cellular state switches.^1^ Such plasticity enables cancer cells to adapt and thrive under environmental pressures including immune surveillance, nutrient deprivation, and therapy.^2^^,^^3^^,^^4^ Across tumor types, co-opting of common developmental (fetal) lineage programs often underlie tumor progression and drug resistance.^5^^,^^6^^,^^7^^,^^8^^,^^9^^,^^10^^,^^11^^,^^12^ A deeper understanding of the interplay between epigenetic, transcriptional, and metabolic plasticity in cell state reprogramming is needed to make transformative progress toward curtailing tumor state transitions and enhancing treatment efficacy.

For many patients with advanced melanoma, systemic targeted and immune therapies have greatly improved prognosis.^13^^,^^14^^,^^15^^,^^16^^,^^17^ However, melanoma cell subpopulations undergoing phenotypic transitions into dedifferentiated stem-like states cause innate or acquired drug resistance and tumor recurrence. In such subpopulations, a transcriptional state resembling that of neural crest (NC) stem cells (NCSCs) and characterized by low activity of melanocyte-inducing transcription factor (MITF) emerges.^12^^,^^18^^,^^19^^,^^20^ This state then becomes enriched after therapy, which is predictive for patient outcomes.^4^^,^^8^^,^^9^^,^^10^^,^^12^^,^^18^^,^^19^^,^^21^^,^^22^^,^^23^^,^^24^^,^^25^ In addition to transcriptional states, metabolic heterogeneity has recently come to the forefront as a mechanism influencing tumor cell plasticity and survival.^26^^,^^27^^,^^28^^,^^29^^,^^30^^,^^31^^,^^32^^,^^33^^,^^34^^,^^35^^,^^36^ While the developmental NC state is known to be highly sensitive to metabolic deficiencies,^37^^,^^38^^,^^39^^,^^40^^,^^41^ we lack an understanding of how metabolic states are coordinated with NC programs in melanoma.

In this study, we discover that the pan-cancer stem cell marker ALDH1A3 is a central regulator of both metabolic and stem cell transcriptional states in melanoma. By tracing acetaldehyde to acetyl-histone H3, we demonstrate it is a source for acetylated histones dependent on ALDH1A3 and link this to transcription of genes regulating NCSC and glucose metabolism. Our findings uncover an actionable, high-dimensional metabolic-transcriptional framework that controls melanoma stem cell plasticity.

## Results

### ALDH1A3^High^ melanomas are enriched for NCSC and glucose metabolic states

When patients with melanoma become resistant to mitogen-activated protein kinase (MAPK) inhibitor therapy, their tumor cells can upregulate stem cell marker expression including ALDH1 enzymes (Figure S1A).^42^^,^^43^^,^^44^ In mouse melanoma models, we find *Aldh1a3* expression is tightly associated with dedifferentiated, NC, and stem cell states, which have been reported to fuel cancer growth in a cellular hierarchy^45^^,^^46^ (Figures S1B and S1C). From these observations, we hypothesized that the ALDH-high metabolic activity of cancer cells is the consequence of a changed transcriptional state that contributes to melanoma stemness and plasticity.

**Figure 1:**
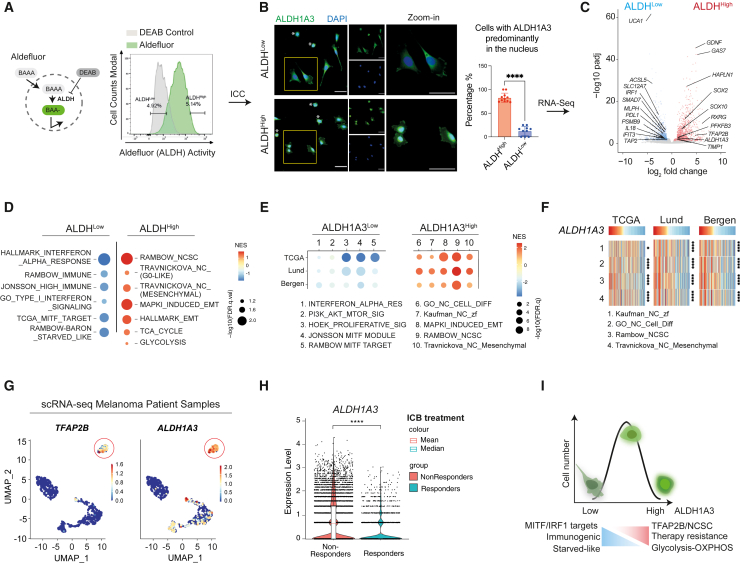
ALDH1A3^High^ melanomas are enriched for NCSC and glucose metabolic states (A) ALDH^High^ and ALDH^Low^ melanoma cell subpopulations. The Aldefluor assay quantifies ALDH activity in live cells by measuring the accumulated fluorescence from converted BODIPY-aminoacetaldehyde (BAAA) to BODIPY-aminoacetate (BAA−). Highest and lowest Aldefluor activity, termed ALDH^High^ and ALDH^Low^ subpopulations, were isolated by FACS. Negative control: diemethylaminobenzaldehyde (DEAB), a pan-ALDH inhibitor. (B) ALDH1A3 cellular localization and levels. Immunocytochemistry (ICC) staining by fluorescence antibody labeling ALDH1A3 in A375 sorted ALDH^High^ and ALDH^Low^ cells. Scale bar, 50 μm. Quantification of cells enriched with nuclear ALDH1A3: experiment repeat *n* = 2, quantified image fields: 11 for ALDH^High^ and 9 for ALDH^Low^, mean ± SD; non-paired Kolmogorov-Smirnov test, ^∗∗∗∗^*p* < 0.0001. Cells showing minimal cytoplastic content outside DAPI-stained regions (indicated with an asterisk [^∗^]) were determined as unfit following FACS and thus excluded from quantification. (C) Differential gene expression in ALDH^High^ and ALDH^Low^ melanoma cells. Volcano plot showing differentially expressed genes analyzed from DESeq2. *n* = 3, fold change >1, adjusted *p* <0.05. See also Table S1. (D and E) Gene set enrichment analysis in melanoma cells and patient samples. Dot plot of pathway analysis showing significantly enriched terms (D) in ALDH^High^ and ALDH^Low^ cells and (E) in ALDH1A3^High^ and ALDH1A3^Low^ patient samples. Dot sizes represent -log_10_ FDR q value (weighted Kolmogorov-Smirnov test) and colors indicate normalized enrichment score (NES). Patient groups are defined in Figure S1G. See also Tables S1 and S2. (F) Heatmap of NCSC gene sets variation analysis (GVSA) score from patient samples ranked by the *ALDH1A3* level. Melanoma patient samples from The Cancer Genome Atlas (TCGA), Lund, and Bergen cohorts unanimously showed positive correlation between ALDH1A3 and NCSC gene sets as annotated. ^∗^*p* < 0.05, ^∗∗∗∗^*p* < 0.0001, Spearman’s rank correlation critical probability exact (p) value. (G) *ALDH1A3* and *TFAP2B* scRNA-seq cluster in metastatic patient samples. Uniform manifold approximation and projection (UMAP) feature plot showing expression level of *ALDH1A3* and *TFAP2B* in scRNA-seq of re-analyzed patient samples of metastatic melanomas.^47^ Red circle highlights an *ALDH1A3* and *TFAP2B* cluster. (H) ALDH1A3 levels are significantly higher in ICB non-responders. scRNA-seq data from approximately 14, 200 malignant cells from Pozniak et al., 2024^48^ were interrogated for *ALDH1A3* expression and early response to ICB. ∗∗∗∗*p* < 0.0001, Wilcoxon signed-rank test. (I) Schematic of ALDH1A3 activity stratified states. High levels of ALDH1A3 Aldefluor activity are associated with nuclear localization and a high glucose metabolism and TFAP2B-NC stem cell dual state. Low levels of ALDH1A3 Aldefluor activity are associated with a differentiated, immunogenic, and starved-like state. See also Figure S1 and Tables S1 and S2.

To investigate this, we considered that ALDH-high (ALDH^High^) activity is heterogeneous in human cell lines (Figure S1D), and that the predominant ALDH activity in melanoma cell line A375 is due to ALDH1A3.^42^ Thus, we sorted A375 cells for the highest and lowest ALDH activity, termed ALDH^High^ and ALDH^Low^, using Aldefluor (a fluorescent amino acetaldehyde) (Figure 1A). As demonstrated previously, these sorted ALDH^High^ cells have increased tumor-initiating potential.^42^^,^^44^ When we validated ALDH1A3 expression by immunocytochemistry (ICC), we were intrigued to see that ALDH1A3 was predominantly expressed in the cytosol in ALDH^Low^ cells but was enriched in the nucleus of sorted ALDH^High^ cells (Figure 1B).

We then conducted RNA sequencing (RNA-seq) of sorted ALDH^High^ and ALDH^Low^ cells and identified high expression of genes associated with dedifferentiated, NC, and stem cell states (including *SOX2*, *SOX10*, *GAS7*, *GDNF*, *RXRG*, and *TFAP2B*) in ALDH^High^ cells (Figures 1C, 1D, and S1E; Table S1 and S2). These genes were also identified as the top marker genes for a less-differentiated state as measured by CytoTRACE in murine melanoma (Figure S1F). This molecular profile resembles a cell state present in drug-resistant melanomas that is predictive of poor outcomes for patients treated with both targeted and immunotherapy.^9^^,^^10^^,^^49^

In contrast, ALDH^Low^ cells were enriched for interferon regulatory factor 1 (IRF1) target genes (*IFIT3*, *IL18*, *PDL1*), proinflammatory genes (*TAP2*, *PSMB9*, *SMAD7*) as well as MITF target pigmentation (differentiation) genes (*MLPH*) (Figures 1C and 1D; Table S1). Metabolic-transcriptional signatures were also highly distinct in ALDH^High^ and ALDH^Low^ cell populations, with glycolysis pathway genes (*PFKFB3*, *PDHX*) enriched in ALDH^High^ cells, whereas starvation response-related fatty acid metabolism genes (*ACSL5*, *SLC12A7*) were enriched in ALDH^Low^ cells (Figure 1D). Taken together, the ALDH1A3^High^ state has features of both NCSC and high glucose metabolism, while ALDH1A3^Low^ has proinflammatory and differentiation features.^20^^,^^37^^,^^50^^,^^51^

Next, we asked if the ALDH1A3-enriched state is present in human patient samples. We ranked patient samples from available datasets based on *ALDH1A3* RNA levels, and then selected *ALDH1A3-high* and *ALDH1A3-low* patients from each cohort; top and bottom 10% for TCGA^52^ and Lund (primary and metastatic melanomas),^53^ and top and bottom 25% for Bergen dataset (stage IV melanomas)^54^ (Figure S1G). Gene expression-based melanoma subtype consensus has previously been established from these cohorts, which successfully stratified prognosis independent of oncogenic genotype.^55^ In our case, for the *ALDH1A3-high* samples, we found that the NCSC state as well as mesenchymal state (epithelial to mesenchymal transition [EMT]) were enriched (Figures 1E, 1F, and S1H; Tables S1 and S2). Analysis of a fourth independent patient sample dataset revealed a direct positive correlation between *ALDH1A3* expression and NCSC transcriptional signatures (Figure S1I).

We were particularly intrigued to see a strong association between *ALDH1A3* and *TFAP2B*, given that we have recently shown *tfap2b* marks an adult multipotent melanocyte stem cell population in zebrafish^46^ (Figures 1C, S1E, and S1F). The *ALDH1A3-TFAP2B* association was isoform specific: across the entire ALDH family, *TFAP2B* was only positively correlated with *ALDH1A3* (Figure S1J). In contrast, *TFAP2A*, a related family member expressed in proliferative melanoma that shares near-identical binding motif with TFAP2B (Figure S1K), was negatively correlated with *ALDH1A3* (Figure S1L). Given these data, we re-analyzed single-cell RNA-seq (scRNA-seq) data from 15 patient melanoma samples^47^ and found that *ALDH1A3* and *TFAP2B* are co-expressed in a distinct cell cluster present in five out of 15 samples of varied mutation subtypes, indicating that there are enough cells in this state to form a cluster and that this cluster does not simply come from a single patient or genotype (Figures 1G and S1M). Thus, in five independent melanoma patient datasets, *ALDH1A3* expression correlates with *TFAP2-NCSC* expression.

In contrast to *ALDH-high* melanomas, and in agreement with our RNA-seq analysis (Figure 1D), *ALDH1A3-low* samples were enriched in proinflammatory gene signatures and MITF pigmentation target genes (Figures 1E; S1H, and S1L). Proinflammation signatures have predictive value of the immune checkpoint blockade (ICB) response, which has greatly improved patient outcomes for advanced-stage melanoma.^56^ We addressed how *ALDH1A3* expression levels correlate with patient response to ICB. We analyzed scRNA-seq of >14,000 melanoma cells from treatment-naive patients with stage III/IV melanoma who later received ICB (nivolumab or ipilimumab and nivolumab) and we found *ALDH1A3*-high expression associated with “non-responders,” while *ALDH1A3*-low expression was associated with "responders" to ICB (Figure 1H).

Collectively, our data support a model in which high ALDH1A3 metabolic activity segregates with transcriptional activation of *TFAP2B-NCSC*-driven developmental stem cell program, high expression of glucose metabolism genes, and resistance to both targeted and ICB therapy, while low-ALDH1A3 metabolic states have features of melanocyte differentiation, low glucose metabolism, and robust immunogenicity (Figure 1I).

### TFAP2B promotes stemness and dedifferentiation in ALDH1A3^High^ melanoma cells

**Figure 2:**
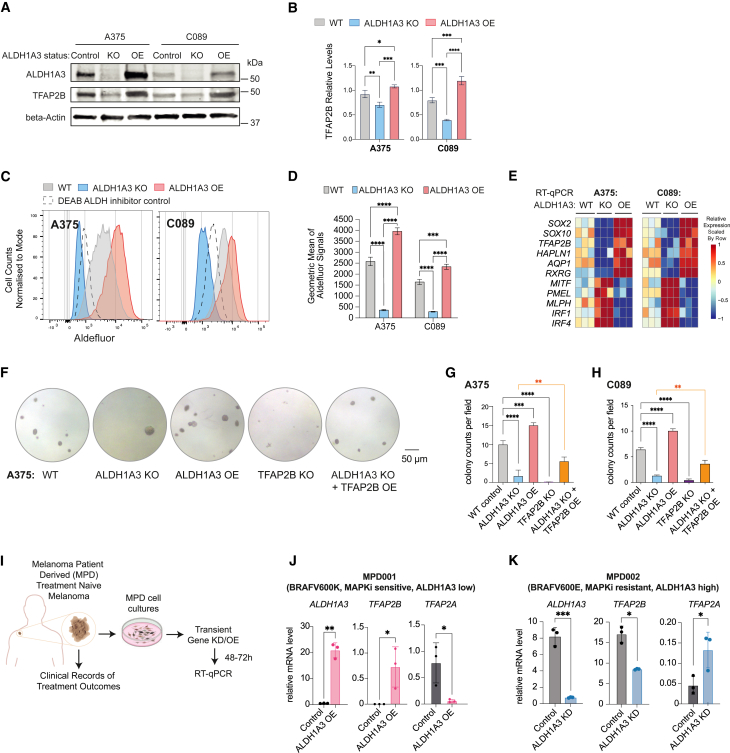
TFAP2B promotes stemness and dedifferentiation in ALDH1A3^High^ melanoma cells (A and B) TFAP2B protein levels are dependent on ALDH1A3. Western blot and quantification of ALDH1A3 and TFAP2B protein expression in ALDH1A3 control, KO, and OE cells. *n* = 3, mean ± SD; one-way ANOVA with Tukey’s test for multiple comparisons. (C and D) ALDH1A3 melanoma cell models. Aldefluor activity and quantification in *ALDH1A3* KO, OE, and vehicle control cells (A375 and C089 cells). *n* = 3, mean ± SD; one-way ANOVA with Tukey’s test correction for multiple comparisons. (E) The ALDH1A3-NC stem cell (NCSC) gene signature. RT-qPCR results of NCSC signature genes in control versus *ALDH1A3* KO and OE cells (A375 and C089 cells, three bio-replicants with three technical replicates each). (F–H) *TFAP2B* rescues *ALDH1A3* activity in colony assays. Representative images and quantification of colonies formed by A375 cell control, *ALDH1A3* KO, *ALDH1A3* OE, *TFAP2B* KO, and combined *ALDH1A3* KO with *TFAP2B* OE conditions. Quantification on both A375 and C089 cells across each condition group, *n* = 3 per condition, mean ± SD one-way ANOVA with Tukey’s test for multiple comparisons. (I) Schematics of the establishment of low-passage melanoma patient-derived (MPD) cells and experimental design for molecular profiling of ALDH1A3 target genes. (J and K) *ALDH1A3* OE promotes *TFAP2B* expression in low-passage patient cells. MPD 001 and 002 cells are ALDH1A3 low, MAPKi sensitive, ALDH1A3 high, and MAPKi resistant, respectively. RT-qPCR of *ALDH1A3*, *TFAP2A*, *TFAP2B* expression in control cells and cells over-expressing *ALDH1A3*. *n* = 3, mean ± SD; multiple non-paired t test corrected with Holm-Sidak’s method. ^∗^*p* < 0.05; ^∗∗^*p* < 0.01; ^∗∗∗^*p* < 0.001; ^∗∗∗∗^*p* < 0.0001. See also Figure S2.

We next asked whether ALDH-associated melanoma stem cell phenotypes are directly regulated by TFAP2B. To do this, we selected two cutaneous melanoma cell lines, A375 and C089, both bearing wild-type (WT) p53 and BRAF(V600E) mutations.^42^^,^^57^ *ALDH1A3* and *TFAP2B* are both expressed at high levels in A375 and at moderate levels in C089 (Figure 2A). Next, we engineered these two lines to knock out or overexpress *ALDH1A3* (Figure S2A). By western blot, we validated that *ALDH1A3* knockout (KO) led to reduced TFAP2B, while *ALDH1A3* overexpression (OE) upregulated TFAP2B (Figures 2A and 2B). Using Aldefluor, we validated *ALDH1A3* KO and OE has significantly shifted ALDH activity toward low and high states, respectively (Figures 2C and 2D). Consistently, RT-qPCR revealed that *ALDH1A3* KO led to reduced *TFAP2B-NCSC* gene expression, concomitant with increased expression of *IRF1* and *MITF* target genes in both cell lines, while this response was reversed in cells that overexpressed *ALDH1A3* (Figure 2E). Taken together, these data support that ALDH1A3 is a direct regulator of the *TFAP2B-NCSC* state.

Next, to test the phenotypic response to altering *ALDH1A3* and *TFAP2B*, we found that *ALDH1A3* KO led to decreased colony formation while *ALDH1A3* OE increased colony formation in both A375 and C089 (Figures 2F–2H), consistent with the pan-cancer effect of ALDH activity in cancer stem-like states.^42^^,^^43^^,^^58^ Remarkably, *TFAP2B* KO phenocopied *ALDH1A3* deficiency in colony assays (Figures 2F–2H). We were unable to assess the impact of *TFAP2B* OE alone in colony assays due to neuronal-like differentiation, consistent with TFAP2B being a powerful NC development regulator. However, in *ALDH1A3* KO cells, *TFAP2B* OE partially rescued the stemness deficiency, leading to enhanced colony-formation capacity in both *ALDH1A3* and *TFAP2B* KO cells (Figures 2F–2H and S2B). These data indicate that TFAP2B is a downstream mediator of ALDH1A3 that sustains cancer stemness in melanoma.

Next, we set out to test if ALDH1A3 mediates *TFAP2B* expression in patient samples. We obtained low-passage human melanoma cells with associated clinical treatment response data, termed melanoma patient-derived (MPD) cells. With this new resource, we employed MPD001 and MPD002 cells from patients with BRAFV600-mutant melanoma, with relatively low and very high levels of ALDH1A3, respectively (Figures 2I and S2C). The clinical records show that the patient’s disease for MPD001 donor was sensitive initially but then continued to progress on immune therapy (ipilimumab) as well as targeted therapies (vemurafenib), while the patient’s disease for MPD002 was innately resistant to vemurafenib as well as ipilimumab. We found that *TFAP2B* was upregulated in MPD001 *ALDH1A3* OE cells, whereas melanocyte differentiation gene *TFAP2A* was downregulated (Figure 2J). Moreover, these changes upon *ALDH1A3* OE segregated with an observable loss of pigmentation, indicative of melanocyte de-differentiation into a stem cell-like state (Figure S2D). In contrast, *ALDH1A3* knockdown (KD) in MPD002 cells led to decreased *TFAP2B*, whereas *TFAP2A* was increased (Figure 2K). Thus, we conclude that *ALDH1A3* is sufficient to promote *TFAP2B* gene expression.

### ALDH1A3^High^ cells use glucose while ALDH1A3^Low^ cells rely on acetate for acetyl-coenzyme A production

**Figure 3:**
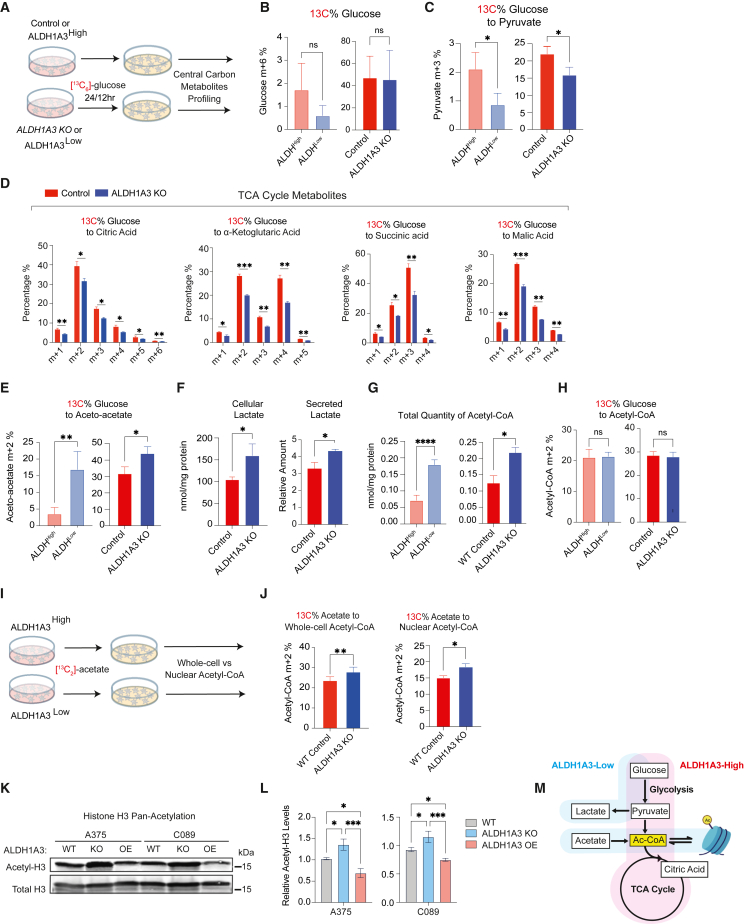
ALDH1A3^High^ cells use glucose while ALDH1A3^Low^ cells rely on acetate for acetyl-CoA production (A–E) (A) Schematic of ^13^C_6_ glucose tracing experiment. Central carbon metabolites were profiled by UPLC-MRM/MS (ultra-performance liquid chromatography electrospray ionization-tandem mass spectrometry in multiple reactions monitoring [MRM] mode) in (B). Glucose (C) and pyruvate (D) TCA cycle metabolites: citrate, alpha-ketoglutarate, succinic acid, malic acid, and (E) aceto-acetate (*n* = 3 for WT vs. ALDH1A3 KO cells; *n* = 5 for ALDH^High^ vs. ALDH^Low^ cells. Multiple paired t test corrected with Holm-Sidak’s method.). (F) *ALDH1A3* KO cells generate and secrete lactate. Intracellular lactate measured by targeted UPLC-MRM/MS (*n* = 3, multiple paired t test corrected with Holm-Sidak’s method). Secreted lactate levels were measured by a colorimetric lactate assay kit and normalized to standards (*n* = 3, unpaired student t test). (G and H) *ALDH1A3* KO cells generate more acetyl-CoA but not from glucose. (G) Intracellular glucose without 13C_6_ labeling and (H) total acetyl-CoA measured by targeted UPLC-MRM/MS (*n* = 3, multiple paired t test corrected with Holm-Sidak’s method). (I and J) Schematic of ^13^C_2_ acetate tracing experiment. ^13^C_2_ acetate tracing experiment designed for ^13^C-incoporation profiling by high-pressure liquid chromatography (HPLC) in (J) whole-cell and nuclear acetyl-CoA. *n* = 5, two-way ANOVA corrected with Holm-Sidak’s method. (K and L) Western blot analyses of pan-acetyl-histone H3 (acetyl-K9 + K14 + K18 + K23 + K27) and total histone H3 protein levels in lysates of A375 and C089 cell lines with vehicle control (WT), *ALDH1A3* KO, or *ALDH1A3* OE, respectively, with total histone H3 probed as loading control and (L) quantification (*n* = 3, mean ± SD; one-way ANOVA with Tukey’s correction for multiple comparisons.). (M) Schematic of ALDH1A3 and metabolic states. ALDH1A3 acts through two different sources of acetyl (Ac)-CoA production: high glucose flux generates high levels of pyruvate, leading to Ac-CoA in ALDH^High^ cells, while ALDH^Low^ cells preferentially uptake acetate as an Ac-CoA source. ^∗^*p* < 0.05; ^∗∗^*p* < 0.01; ^∗∗∗^*p* < 0.001; ^∗∗∗∗^*p* < 0.0001. See also Figures S3 and S4 and Table S3.

Next, we sought to explore relationships between ALDH1A3 function and the metabolic states informed by RNA-seq in cells and in patients (Figure 1). To this end, we traced ^13^C_6_-labeled glucose in two A375-based melanoma cell models: (1) WT control versus *ALDH1A3* KO cells following 24-h labeling; and (2) following 12-h labeling, non-engineered cells sorted for ALDH1A3^High^ and ALDH1A3^Low^ (Figure 3A; Table S3). We did not find any difference in ^13^C_6_-glucose uptake between cells having different levels of *ALDH1A3* (Figure 3B), despite the increased expression of glycolysis genes in ALDH1A3^High^ cells determined by RNA-seq analysis. Instead, we observed significantly higher glucose-derived carbon flux in cells with high ALDH1A3, as shown by an increase in ^13^C-labeled carbon in pyruvate and tricarboxylic acid (TCA) cycle intermediates, including citric acid, α-ketoglutaric acid, succinic acid, and malic acid (Figures 3C and 3D). Conversely, in *ALDH1A3* KO and ALDH1A3^Low^ cells, glucose-derived carbon was converted into ketone bodies such as aceto-acetate, a metabolite synthesized from acetyl-coenzyme A (CoA), reflecting a starvation-like metabolic state (Figure 3E). Despite the lower glycolytic flux, *ALDH1A3* KO cells produced higher levels of lactate both in cells and secreted into the culture medium (Figure 3F). These data indicate that ALDH1A3^High^ cells are primed to execute glycolysis and oxidative phosphorylation (OXPHOS), whereas ALDH1A3^Low^ cells exist in a starvation-like state and secrete lactate. Consequently, we reasoned these states exert differential metabolic and epigenetic effects on surrounding cancer and stromal cells.^59^^,^^60^

Acetyl-CoA, an acetyl group donor in biochemical reactions, is generated from pyruvate through glycolysis or through oxidation of long-chain fatty acids or certain amino acids, and its availability is known to shape both metabolic processes as well as epigenetic regulation.^61^ Unexpectedly, we found that total levels of acetyl-CoA increased in ALDH1A3^Low^ cells despite decreased glycolysis and reduced TCA cycle flux (Figure 3G). This increase of total acetyl-CoA was not derived from a glucose source, as indicated by ^13^C_6_ glucose tracing (Figure 3H). Given that cancer cells under metabolic stress can support as much as half of their lipid synthesis by using acetate as a substrate,^62^ we hypothesized that ALDH1A3^Low^ melanoma cells supplement their carbon source by switching from glucose to acetate. When we traced ^13^C_2_-acetate incorporation into acetyl-CoA (Figure 3I), we found that *ALDH1A3* KO cells had a higher percentage of acetate-derived acetyl-CoA relative to control cells, both within the whole cell and in the nuclear fraction (Figure 3J).

To address the potential link between metabolic processes and chromatin modification, we found that the increase in acetyl-CoA in *ALDH1A3* KO and sorted ALDH^Low^ cells was also concomitant with a significant increase in acetyl-histone H3 (∼30%) (Figures 3K, 3L, and S3A), while ALDH1A3 OE led to a ∼15% decrease (Figures 3K, 3L, and S3A). Thus, we conclude that cells with high levels of ALDH1A3 rapidly metabolize glucose to generate pyruvate, while cells with low ALDH1A3 activity preferentially use acetate as a carbon source for acetyl-CoA (Figure 3M).

### Pyruvate-derived acetaldehyde serves as an acetyl source for histone H3-acetylation

**Figure 4:**
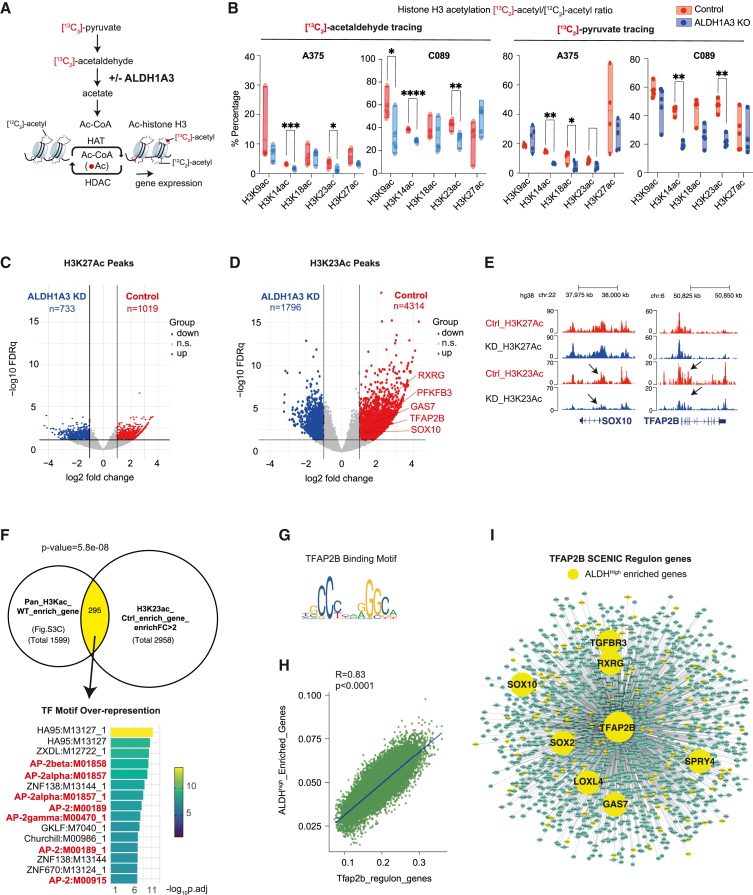
Pyruvate-derived acetaldehyde serves as an acetyl source for histone H3-acetylation (A) Schematic summarizing the acetyl group transfer from pyruvate to histone mediated by ALDH1A3 metabolizing acetaldehyde, with the experiment design that ^13^C_2_-acetaldehyde and/or ^13^C_3_-pyruvate-derived acetyl groups can be traced to histone acetylation. (B) Pyruvate-derived acetaldehyde is an acetyl source across multiple lysine residues on histone H3. ^13^C_2_-acetyl groups were traced from acetaldehyde into lysine acetylation at K9, K14, K18, K23, and K27, measured by HPLC-MS/MS. *ALDH1A3* KO cells treated with ^13^C_2_-acetaldehyde showed fewer ^13^C_2_-acetyl groups at K9, K14, and K23 residues. Similar differential patterns of ^13^C_2_-acetyl group incorporation at histone H3 were observed when cells were treated with ^13^C_3_ sodium pyruvate. *n* = 5 per cell line; *p* values by two-way ANOVA with Sidak’s multiple comparisons test. (C and D) Volcano plot of differentially acetylated histone H3K27 peaks (C) and H3K23 peaks (D) from CUT&TAG in MPD002 control and ALDH1A3 KD cells (highlighted by red and blue respectively, fold change >2, FDR q <0.05). Representative NCSC gene-related peaks are annotated in the control enriched H3K23 peaks. (E) Gene tracks of acetylated histone H3K27 and H3K23 peaks in MPD002 vehicle control versus *ALDH1A3* KD cells at representative NCSC genes. No significant changes are present in H3K27, while the significant change in H3K23 acetylation is observed. (F) Venn diagram of overlapping genes between (upper left) genes enriched in A375 control cells versus *ALDH1A3* KO cells mapped from total histone H3 acetylation and (upper right) MPD002 control cells versus ALDH1A3 knockdown cells mapped from histone H3K23 acetylation. *p* values by Fisher’s exact test. Transcription factor binding motif over-representation analysis of the Venn diagram overlapping genes (lower panel) showed significant enrichment of AP-2 binding motif, with enrichment score by g:Profiler (e111_eg58_p18_30541362) with g:SCS multiple testing correction method applying statistical significance threshold of 0.05.^64^ (G) Binding motif of human TFAP2B from JASPAR database. (H) Scatterplot shows the correlation of ALDH^High^ enriched gene signature and Tfap2b regulon activities (AUCell score) in murine melanoma cells (*NRAS*^*Q61K/°*^*;Ink4a*^*−/−*^).^46^ (I) SCENIC-inferred Tfap2b regulatory network using a murine mouse scRNA-seq dataset,^46^ with Tfap2b target genes in green and ALDH^High^ enriched Tfap2b target genes highlighted in yellow. Tfap2b regulon genes were mapped to human homologs to allow comparison and visualization. See also Figures S3 and S4 and Tables S4 and S5.

In human cells, pyruvate dehydrogenase (PDH) can generate acetaldehyde, an ALDH substrate, from pyruvate.^63^ We investigated if this pyruvate-derived acetaldehyde pool could serve as a source of histone H3 acetyl modification in melanoma. To answer this question, we traced ^13^C_2_-labeled acetaldehyde and performed histone proteomic analyses (Figure 4A). Indeed, in our two independent melanoma cell lines (control vs. *ALDH1A3* KO), we detected higher levels of ^13^C_2_-labeled acetylated histone H3 proteins in cells with high ALDH1A3 activity, with the incorporation at H3K14ac and H3K23ac especially responsive to ALDH1A3 levels (Figure 4B, left panel). This demonstrates that acetaldehyde can be a direct source for histone H3 acetylation.

Next, we traced ^13^C_3_-labeled pyruvate to histones, and again consistently detected higher percentage of ^13^C-labeled acetylated histone H3 proteins in cells with high ALDH1A3 activity (Figure 4B, right panel). This observation supports a model in which pyruvate can serve as the source of acetaldehyde and subsequently acetate for acetyl-CoA production.

### ALDH1A3 alters selective histone H3 acetylation in the genome

To better understand the significance of ALDH1A3 dependent acetyl-histone H3, we considered that selective histone acetylation mechanisms can regulate specific gene expression, including glucose metabolism enzymes,^65^^,^^66^ and that global histone repositories serve as reservoirs for acetyl groups.^67^ We hypothesized that pyruvate-derived acetaldehyde serves as a local source of acetyl-CoA for histone acetylation at specific gene loci to generate a cancer stem cell-like state. Conversely, in the low-ALDH1A3 state, acetate-derived acetyl-CoA is deposited on chromatin as a reservoir,^67^ serving as a rapid source for acetyl-CoA and as a buffer for intracellular pH.^68^^,^^69^

To determine how ALDH1A3-dependent histone H3 acetylation is deposited on chromatin, we first performed quantitative acetyl-histone H3 chromatin immunoprecipitation (ChIP)-seq using an antibody against pan-histone H3 acetylated sites (K9 + K14 + K18 + K23 + K27) (Methods S1; Figures S3 and S4). In control cells, the enriched acetyl-histone H3 peaks were clustering around transcription start sites (TSSs), especially within 1 kb of promoters, whereas, in *ALDH1A3* KO cells, the enriched acetyl-histone H3 peaks were broadly dispersed throughout the genome, and particularly spreading into the distal intergenic region and intronic regions (Figures S3D and S3E). Next, we found that acetyl-histone H3 sites enriched in the ALDH1A3^High^ state were present in NCSC and glucose metabolism pathway genes, while, conversely, the loss of *ALDH1A3* was associated with a broadly dispersed acetyl-histone H3 landscape in which subsets of elevated acetyl-histone H3 peaks were associated with proinflammatory genes (Figures S4G and S4H; Table S4).

While consistent with the RNA expression patterns that we identified earlier, the use of the pan-histone acetylation antibody that recognizes five different marks could be masking the effect of site-selective acetylation events. To address this, we performed CUT&TAG analysis in MPD002 control and *ALDH1A3* KD cells using individual antibodies selective in H3K23ac and H3K27ac, with H3K23ac indicated as dependent on ALDH1A3 from 13C-labeled acetaldehyde and pyruvate tracing and H3K27ac not affected. We found limited differential peaks between WT and *ALDH1A3* KD groups for H3K27ac, with much stronger differential effects for H3K23ac (Figures 4C and 4D). More importantly, by mapping the H3K23ac-enriched peaks in the control compared to *ALDH1A3* KD, we again identified NCSC genes, including TFAP2B and SOX10 (Figure 4E). Further, by comparing the total enriched H3 acetylation peak target genes in A375 (control versus *ALDH1A3* KO) with the enriched H3K23ac peak target genes in MPD002 (control versus *ALDH1A3* KD), we found the overlapping target genes are over-represented for TFAP2 transcription factor (AP-2) motif (Figures 4F and 4G; Table S5).

The discovery of the TFAP2 motif supports a likely TFAP2B feedforward loop, acting both as a direct target and mediator of ALDH1A3-dependent gene expression and thereby establishing and sustaining the ALDH1A3^High^ NC identity. To test our prediction, we employed gene-regulatory network inference (SCENIC)^70^ and identified a melanoma derived *Tfap2b* regulon (Table S5). When comparing AUCell expression scores of the *Tfap2b* regulon with our ALDH^High^ enriched gene set across 16,700 single melanoma cells, originating from five primary mouse murine lesions,^46^ we detected significant co-expression of both transcriptional programs (Figure 4H). When we intersected Tfap2b regulon genes with ALDH^High^ enriched genes, we found a striking overlap that included both NC and stem cell genes such as *SOX2*, *SOX10*, *SPRY4*, and *RXRG* (Figures 4I; Table S5).

### ALDH1A3 forms a predicted complex with ACSS2

**Figure 5:**
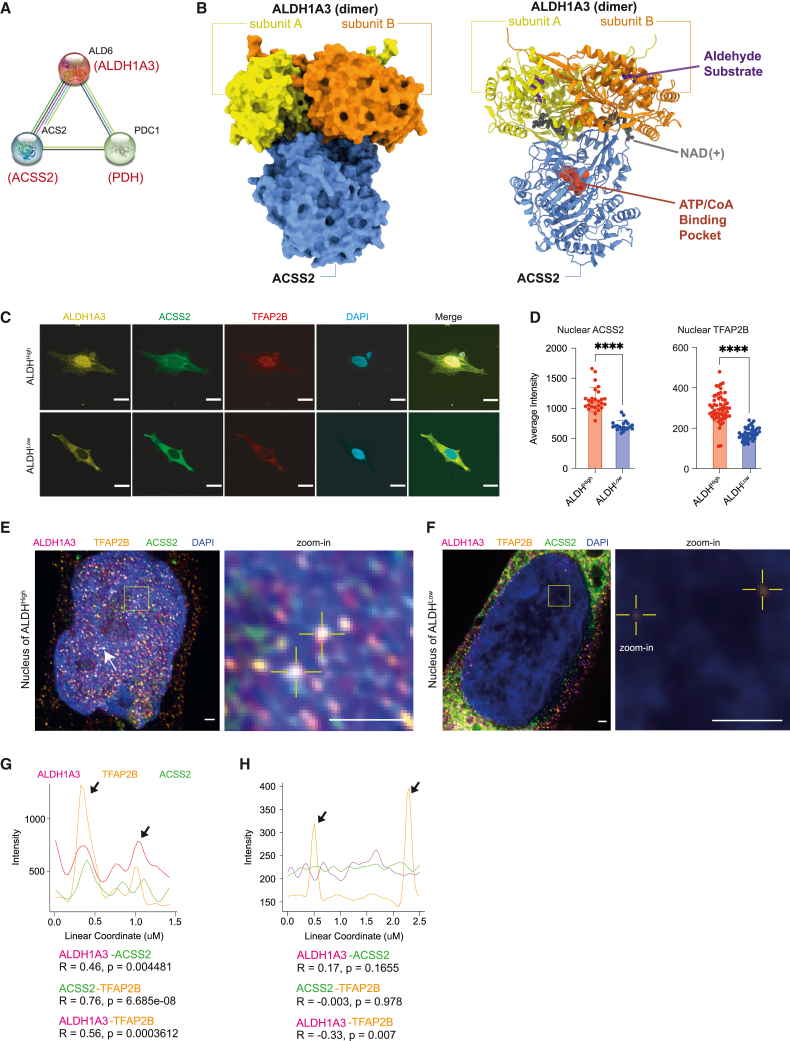
ALDH1A3 forms a predicted complex with ACSS2 (A) ALDH1A3 functional association. STRING functional protein association analysis between yeast Ald6, Acs2, and Pdc1, orthologous to human ALDH1A3, ACSS2, and PDH. (B) AlphaFold Multimer modeling of human ALDH1A3 and ACSS2. Proteins visualized as a complex in surface model (left) and ribbon model (right). The binding sites of an aldehyde substrate (retinaldehyde) and co-factor (NAD+) were created by structural alignment of the AlphaFold-Monomer predicted structure to PDB: 5fhz, a published tetramer ALDH1A3 structure. The ATP/CoA binding pocket of ACSS2 was visualized by structural alignment of AlphaFill-optimized protein-ligand complexes (ATP donor, PDB: 5k8f. CoA donor, PDB: 3gpc). (C and D) Subcellular expression of ALDH1A3, ACSS2, and TFAP2B. (C) ICC staining by fluorescence antibody probing ALDH1A3 (yellow), ACSS2 (green), and TFAP2B (red) in sorted A375 ALDH^High^ and ALDH^Low^ cells. DAPI (blue). Scale bar, 10 μm. (D) Fluorescence signal intensity quantification of nuclear TFAP2B and ACSS2 in ICC images. 2 biological repeat experiments, *n* = 55 quantified single cells for ALDH^High^ TFAP2B, *n* = 46 single cells for ALDH^Low^ TFAP2B; *n* = 26 single cells for ALDH^High^ ACSS2, *n* = 20 single cells for ALDH^Low^ ACSS2 (represented as individual dots), mean ± SD, unpaired non-parametric Kolmogorov-Smirnov test. ^∗∗∗∗^*p* < 0.0001. (E and F) ALDH1A3, ACSS2, and TFAP2B co-localize in the nucleus. Structured illumination microscopy (SIM) of ALDH1A3 (magenta), TFAP2B (orange), ACSS2 (green), and DAPI (blue) in A375 melanoma FACS-sorted (E) ALDH^High^ cells and (F) ALDH^Low^ cells. Scale bar, 1 μm. Complex co-localization signals were abundant (arrows) and are indicated as highlighted dots in the zoomed image in (E). Low signals of nuclear TFAP2B are highlighted in zoomed image in (F). (G) Intensity plot profiles of the line scan across two co-localization hotspots in zoomed image of (E). Signal overlap peaks are indicated by arrows. Pearson correlation test. (H) Intensity plot profile of the line scan across the two TFAP2B signal spots in zoomed image of (F). TFAP2B signal peaks are indicated by arrows, where no ALDH1A3 or ACSS2 signals are present. Pearson correlation test. See also Figure S5.

Nutrition, cellular metabolism, and transcription are intimately linked with epigenetic control of gene expression.^71^ When we analyzed the ALDH1A3 protein interaction network database via STRING,^72^ we noted that acetyl-CoA synthetase 2 (Acs2) and pyruvate decarboxylase (Pdc) are interacting partners of aldehyde dehydrogenase (Ald6; ortholog of ALDH1) in yeast (*Saccharomyces cerevisiae*)^73^ (Figure 5A). This was intriguing as, in neuronal stem cell differentiation and memory formation in mammals, ACSS2 was reported to generate acetyl-CoA “on-demand” from chromatin-bound acetate associated with selective histone acetylation and gene expression.^74^^,^^75^

Based on this, we used AlphaFold Multimer^76^ to predict interactions between yeast Acs2 and Ald6 and between their human homologs ACSS2 and ALDH1A3 (Figures 5B and S5A). This approach yielded complexes for both the yeast and human pairs with moderate confidence (45% and 47%, respectively). In addition, we identified strong interface conservation between Acs2 and ACSS2 (Pearson correlation of 0.61 between buried surface area of homologous residues, *p* = 1.7 × 10^−66^), with Acs2/ACSS2 binding at the perimeter of the Ald6/ALDH1A3 dimerization interface in a 1:2 stoichiometry. This binding mode ensures accessibility of the Acs2/ACSS2 binding pockets for CoA and ATP and the Ald6/ALDH1A3 substrate pockets for NAD and aldehyde. Thus, both the predicted yeast and human complexes are consistent with an ACSS2-ALDH1A3 enzymatic partnership.

Next, using immunofluorescence staining, we found that ACSS2, along with ALDH1A3 and TFAP2B, was localized in the nucleus of sorted ALDH1A3^High^ cells but not in ALDH1A3^Low^ cells (Figures 5C and 5D). In the presence of an ACSS2 inhibitor, we found no change in nuclear ALDH1A3, whereas nuclear TFAP2B levels were significantly reduced (Figures S5B and S5C). We propose that the ALDH1A3-ACCS2 enzymatic partnership is not required for ALDH1A3 nuclear localization; however, ALDH1A3-dependent TFAP2B expression and nuclear localization are mediated through ACSS2.

Using super-resolution imaging, we detected abundant nuclear co-localization signals of ALDH1A3-ACSS2-TFAP2B and a significant positive correlation between the linear signal distribution of any two of the three target proteins (Figures 5E–5H). In contrast, we saw minimal nuclear signals of ALDH1A3, ACSS2, or TFAP2B in sorted ALDH^Low^ cells and the nuclear signals captured by super-resolution imaging were not overlapping (Figure 5H). Finally, we tested the potential for interaction *in vivo* by co-immunoprecipitation and confirmed the interaction of ACSS2 with ALDH1A3 in both A375 whole cells and the nuclear department (Figure S5D).

### ALDH1A3 determines ACSS2 binding to NCSC gene loci

**Figure 6:**
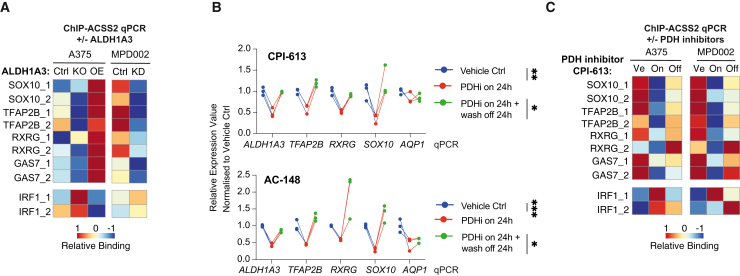
Acetaldehyde serves as an acetyl source for histone H3-acetylation (A) ALDH1A3 directs gene-specific ACSS2 chromatin binding. ACSS2 ChIP-qPCR results in (left) A375 WT control versus *ALDH1A3* KO and *ALDH1A3* OE cells, as well as in (right) MPD002 control versus *ALDH1A3* KD cells (siALDH1A3). *n* = 3 biological replicates each with 3 technical replicates and normalized to immunoglobulin (Ig) G control. (B) RT-qPCR-measured NCSC gene expression change in A375 and MPD002 in response to pyruvate dehydrogenase (PDH) activity change induced by PDH inhibitors (PDHis) CPI-613 (upper) and AC-148 (lower). ^∗^*p* < 0.05; ^∗∗^*p* < 0.01; ^∗∗∗^*p* < 0.001; one-way ANOVA with Sidak’s correction. (C) ALDH1A3-directed gene-specific ACSS2 chromatin binding relies on PDH activity. ACSS2 ChIP-qPCR results in A375 and MPD002 cells with vehicle control (Ve) versus PDH inhibitor CPI-613-treated samples (on, 24 h) and PDH inhibitor wash-off samples (24-h treatment followed by additional 24-h wash-off recovery, off). *n* = 2 biological repeat experiments in both A375 and MPD002 cells, each with 3 technical replicates and normalized to IgG control before heatmap scaling and plotting.

To verify ALDH1A3-ACSS2-dependent acetylated histone loci and target genes, we designed primers targeting the promoter regions of NCSC genes and IRF1 genes and performed ACSS2 ChIP-qPCR in both A375 and MAPKi-resistant MPD002 cells (Figure 6A). ACSS2 ChIP-qPCR revealed higher levels of ACSS2 binding to multiple NCSC gene promoter regions in control comparing to *ALDH1A3* KO/KD cells, while, in contrast, ACSS2 binding was enriched at the *IRF1* promoter in *ALDH1A3* KO/KD cells. This effect was restored upon *ALDH1A3* OE in A375 cells. These data show that ALDH1A3 is required for ACSS2 binding to specific loci to shape the genomic landscape.

### PDH inhibitors alter ACSS2 binding to selective genomic loci

To strengthen our understanding of pyruvate in the ALDH1A3-ACSS2 mechanism, we used two independent PDH inhibitors, the thiamine analogue AC-148^77^ (Data S1) and a commercially available lipoate related PDH inhibitor, CPI-613,^78^ and determined the effect on NCSC gene expression. Addition of either of the PDH inhibitors for 24 h led to decreased NCSC gene expression, followed by restored expression (except *AQP1*) 24 h after wash-off (Figure 6B). Next, we addressed whether PDH inhibition altered the ACSS2 binding to multiple NC gene loci by ChIP-qPCR, in both A375 and MPD002 cells. We found that ACSS2 binding to these regions in control cells was reduced with PDHi treatment and restored upon inhibitor washout (Figure 6C).

We propose a model in which ALDH1A3 and ACSS2 cooperate in a metabolic cascade from pyruvate to acetaldehyde to generate a regional chromatin source of acetyl-CoA from nuclear acetaldehyde used to selectively deposit localized histone H3 acetylation and is associated with increased expression of NCSC genes (including *TFAP2B* itself) and glucose metabolism genes.

### ALDH1A3 cells promote drug resistance and disease recurrence *in vivo*

**Figure 7:**
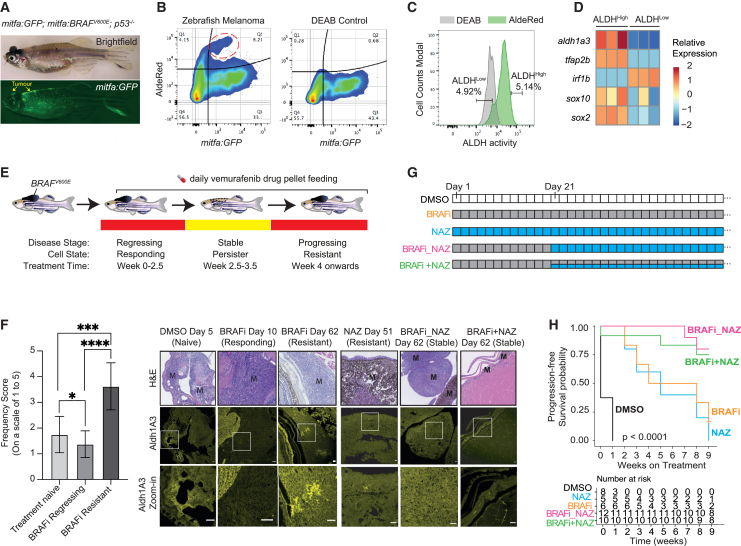
ALDH1A3^High^ subpopulations promote melanoma drug resistance *in vivo* (A) Zebrafish BRAF^V600E^ melanoma model (arrow indicates melanoma). *BRAF*^*V600E*^ and *GFP* are expressed in the melanocyte lineage, by the *mitfa* promoter, and the zebrafish is mutant for p53. (B–D) ALDH^High^ cells with low levels of *mitfa:GFP* in zebrafish melanoma (red circle). DEAB: negative control. (C) FACS of ALDH^High^ and ALDH^Low^ zebrafish melanoma cells. (D) RT-qPCR of *sox2*, *sox10*, and *tfap2b* expression in sorted ALDH^High^ versus ALDH^Low^ zebrafish melanoma cells (*n* = 3 bio-replicates each with 3 technical replicates, multiple paired t test corrected with Holm-Sidak’s method). (E and F) Aldh1a3 in zebrafish model of BRAF inhibitor regression and recurrent disease. (E) *BRAF*^*V600E*^*p53* mutant zebrafish with melanomas were fed with 200 mg/kg/day vemurafenib-containing food pellets leading to melanoma regression, drug resistance. and disease recurrence. (F) Aldh1a3 IHC in DMSO, BRAFi, and/or NAZ treatment-responding disease and in drug-resistant recurrent disease shows Aldh1a3 on-target efficacy of nifuroxazide. One-way ANOVA with Tukey’s test correction for multiple comparisons. Scale bar = 25 μm. ^∗^*p* < 0.05; ^∗∗∗^*p* < 0.001; ^∗∗∗∗^*p* < 0.0001. (G and H) Zebrafish combination drug trial to target ALDH^High^ cells. (G) Long-term drug-pellet treatment design. (H) Kaplan-Meier survival curves of zebrafish melanomas under different drug treatment shown in (G). Log rank tests, *p* < 0.001. See also Figure S6.

To evaluate functional relationships between ALDH1A3^High^ cells, melanoma disease progression, and therapy resistance, we employed the widely used BRAF^V600E^ p53 mutant zebrafish melanoma model, in which BRAF inhibitors initially reduce tumor burden followed by drug resistance and recurrent melanoma growth.^79^^,^^80^^,^^81^ Here, we dissected melanomas from BRAF^V600E^ p53 mutant zebrafish that also express GFP in the melanocyte lineage (*mitfa:GFP*), and performed AldeRed analysis (similar to the Aldefluor assay but using a red fluorescent substrate) (Figures 7A and 7B). Through fluorescence-activated cell sorting (FACS), we identified a distinct zebrafish cell population with ALDH^High^ activity that was also low for *mitfa:GFP* (Figure 7B), indicating that both zebrafish and human melanoma ALDH^High^ cells express low levels of *MITF*. Next, we sorted zebrafish melanoma cells with the highest and lowest ALDH activity and performed RT-qPCR (Figures 7C and 7D). In agreement with our findings in human melanoma, we found that *aldh1a3* was the most enriched *ALDH* family isoform in the ALDH^High^ cells (Figure S6A), together with high *tfap2b*, *sox2*, and *sox10*, while melanoma cells with ALDH^Low^ activity expressed *irf1b* (Figure 7D).

Further, mirroring the observation in human patients, we administered vemurafenib (BRAF inhibitor) drug pellets to adult zebrafish with melanomas^81^ and found that clusters of Aldh1a3 cell populations increased in drug-resistant and progressing disease upon immunohistochemistry (IHC) assessment through disease stages (melanoma responding, stable, and progressive disease) (Figures 7E and 7F). Finally, we designed a zebrafish drug trial to test whether killing the Aldh1a3^High^ cells that emerge during BRAF inhibitor (BRAFi) drug resistance would affect disease outcome (Figures 7F and 7G). We administered nifuroxazide (NAZ), a 5-nitrofuran pro-drug that specifically kills ALDH1^High^ cells in human cell lines and mouse xenograft melanoma models.^42^ As expected, zebrafish melanoma responded rapidly to BRAFi treatment but then grew back quickly despite ongoing treatment (Figure S6B). When we administered NAZ treatment alone, we observed a period of stable disease followed by progressive melanoma growth (Figure S6B).

We then tested combinational drug treatment, using the BRAF inhibitors to target the bulk of the tumor and NAZ to target ALDH1^High^ cells: (1) BRAFi followed by NAZ (BRAFi_NAZ) at day 21, and (2) BRAFi treatment alone until day 21, followed by both BRAFi + NAZ (Figure 7G). Our data demonstrate that both combinations of BRAFi plus NAZ significantly improved disease outcomes and probability of survival (Figures 7H and S6B). To investigate whether the improved outcomes are achieved via on-target effect of NAZ, we examined Aldh1a3 levels in NAZ-treated zebrafish melanomas in progressive, drug-resistant disease and found that Aldh1a3 clusters were absent (Figure 7F). Together, these results support a combination therapeutic strategy using BRAF inhibitors to target the tumor bulk and NAZ to eradicate the ALDH1A3-melanoma stem cell pool in the residual disease.

## Discussion

Cell state heterogeneity and plasticity endow genetically identical cancer cells to respond differently to treatment. As a result, diverse resistant cell states emerge in patients, challenging clinical strategies that target limited static states.^82^ Here, we uncover that nuclear ALDH1A3, functioning as a master coordinator of metabolic and transcriptional cell states, fosters stem-like gene expression programs in melanoma by using pyruvate-derived acetaldehyde as an acetyl donor for histones.

We identified a nuclear ALDH1A3-ACSS2 mechanism that controls both a glycolytic gene expression program and a developmental NCSC program mediated by TFAP2B. Mechanistically, nuclear ALDH1A3-ACSS2 directs a glucose-derived pyruvate-acetaldehyde-acetate-acetyl-CoA flux and selectively deposits histone H3 acetylation, including H3K23ac, at genomic loci encoding for glycolysis and TFAP2B-NCSC genes. Cells with low ALDH1A3 preferentially take up acetate to generate a nuclear acetyl-CoA flux, which activates MITF and IRF1 gene programs but is otherwise broadly deposited on the chromatin as an acetate reservoir (Figure S7A).

Critically, we demonstrate that endogenous acetaldehyde is a metabolite utilized by ALDH1A3 to modify chromatin. This functional relationship expands the scope of acetaldehyde metabolism from solely protecting cells from aldehyde macromolecule adducts.^83^^,^^84^ Endogenous and alcohol-derived acetaldehydes, as well as other aldehydes, including formaldehyde, are highly reactive toward DNA, and cells employ a robust two-tier protection mechanism to protect against aldehyde-induced DNA damage.^83^^,^^85^^,^^86^^,^^87^^,^^88^ Tier 1 involves aldehyde detoxification enzymes (ALDH2 and ADH5) followed by tier 2 Fanconi anemia DNA damage repair pathways to repair aldehyde-induced DNA damage.^84^ Gene variants in these pathways in people lead to a loss of protection and directly contribute to increased cancer risks, bone marrow failure, and risks associated with the alcohol-exposed developing fetus.^89^^,^^90^^,^^91^^,^^92^^,^^93^^,^^94^ However, recent evidence also shows exogenous alcohol can serve as an acetyl source for histones in the brain via ACSS2, near genes involved in learning and memory, and in the liver.^95^^,^^96^ Together with our findings presented here, this points to a possible broader role for acetaldehyde as a metabolite source for chromatin regulatory marks. Supporting this intimate relationship between aldehydes and chromatin modification, recent evidence shows that nucleosome demethylation releases formaldehyde^97^ and that histone deacetylase three suppresses endogenous formaldehyde production, the reaction products of which are used in one-carbon (formate) metabolism.^98^^,^^99^ Thus, chromatin may be both modified by aldehydes to shape the regulatory landscape as well as serving as a source of metabolites when required, including acetyl-CoA and formate.

Our data here, along with recent discoveries that formaldehyde metabolism promotes differentiation of primed melanocyte stem cells,^100^ highlights an emerging physiological role for aldehydes as essential metabolites governing stem cell function. Pioneering studies of nuclear condensates show that the spatial clustering of active gene transcription is intimately linked with nuclear protein-metabolite condensate distribution,^101^ providing a possibility that subnuclear ALDH-aldehyde metabolism may exert local control of gene expression or acetate reservoirs. It will be important for future studies to identify the external signals that promote the ALDH1A3^High^ state following drug treatment and address whether these contribute to the cross-resistance mechanisms triggered by targeted therapies and ICB. Transcriptionally, analysis of two independent hemagglutinin (HA)-tagged MITF ChIP sequencing (ChIP-seq) experiments show HA-MITF binds the ALDH1A3 promoter region,^102^^,^^103^ and, given that we find MITF activity is low in ALDH1A3^High^ cells, this suggests that MITF may be a repressor of ALDH1A3 transcription (Figure S7B). Further, it will be important to identify whether aldehydes available from the microenvironment (possibly even through diet or the microbiome) can affect the metabolism-NCSC state axis in melanoma.

In conclusion, we present evidence that ALDH1A3 is a master regulator of a metabolism-NCSC state axis that partners with ACSS2 to modify histone acetylation by locally depositing acetaldehyde-derived acetyl groups. The conceptual framework we present here for melanoma may be broadly applicable to ALDH^High^ cancer stem cell subpopulations in other cancer types, as ALDH isoforms potentially cooperate with lineage-specific master transcription factors (often from developmental lineages) that are co-opted to regulate tumor cell states.^104^

### Limitations of this study

Additional CUT&TAG or ChIP-seq experiments using independent antibodies for each of the H3K-ac marks would clarify how acetaldehyde-selective histone H3 acetylation relates to the chromatin landscape beyond H3K23 and H3K27. Testing for ACSS2 interactions with other ALDH family members would provide more insight into the specificity and wider applicability of ACSS2-ALDH interactions in other biological contexts. Drugs often have more than a single target *in vivo*, and we are not able to determine the contribution of these potential other targets to the melanoma response to NAZ in our experimental model.

## STAR★Methods

### Key resources table

**Table:** 

REAGENT or RESOURCE	SOURCE	IDENTIFIER
**Antibodies**		

Rabbit polyclonal anti-ALDH1A3	Abcam	Cat # ab129815; RRID: AB_2937054
Rabbit polyclonal anti-ALDH1A3	Thermo Fisher Scientific	Cat# PA5-29188; RRID: AB_2546664
Mouse polyclonal anti-TFAP2B	Santa Cruz	Cat # SC-390119x; RRID: AB_2828008
Mouse monoclonal anti-beta Actin	Abcam	Cat # ab8226; RRID: AB_306371
Rabbit polyclonal anti-acetyl-Histone H3	Millipore	Cat# 06–599; RRID: AB_2115283
Mouse monoclonal anti-Histone H3	Abcam	Cat# ab10799; RRID: AB_470239
Rabbit recombinant monoclonal anti-ACSS2	Abcam	Cat # ab133664; RRID: AB_2943489
Rabbit monoclonal anti-ACSS2 (AceCS1)	Cell Signaling Technology	Cat# 3658; RRID: AB_2222710
Rabbit monoclonal anti-ACSS2	Invitrogen	Cat # PA5-26612; RRID: AB_2544112
Acetyl-Histone H3K23Ac antibody Rabbit mAb	Invitrogen	Cat # PA5109818; RRID:AB_2855229
Acetyl-Histone H3 (Lys27) (D5E4) XP® Rabbit mAb	Cell Signaling Technology	Cat # 8173; RRID: AB_2798743
Goat anti Rabbit secondary antibody unconjugated	Abcam	Cat # ab7085; RRID:AB_955982
Rabbit (DA1E) mAb IgG XP® Isotype Control (CUT&RUN)	Cell Signaling Technology	Cat # 66362; RRID:AB_2924329
IRDye 680RD Goat polyclonal anti-Mouse IgG	LI-COR Biosciences	Cat# 925–68070; RRID: AB_2651128
IRDye 800CW Goat polyclonal anti-Rabbit IgG	LI-COR Biosciences	Cat# 925–32211; RRID: AB_2651127
Donkey anti-rabbit conjugated Alexa Fluor 488	Invitrogen	Cat# A-21206; RRID: AB_2535792
Donkey anti-mouse conjugated Alexa Fluor 568	Invitrogen	Cat# A-10037; RRID: AB_2534013
Donkey anti-rabbit conjugated Alexa Fluor 647	Invitrogen	Cat# A-31573; RRID: AB_2536183

**Bacterial and virus strains**		

One Shot™ Stbl3™ Chemically Competent E. coli	Invitrogen	Cat# C737303

**Biological samples**		

Human patient melanoma samples	Manchester Cancer Research Center (MCRC)	Patient samples were collected with MCRC Biobank ethics application #07/H1003/161 + 5 and with approval for the work under MCRC Biobank Access Committee application 13_RIMA_01

**Chemicals, peptides, and recombinant proteins**		

DAPI (for nucleic acid staining)	Sigma-Aldrich	Cat# D9542
7-AAD (7-Aminoactinomycin D)	Eurogentec	Cat# AS-83201
TRIzol™ Reagent	Invitrogen	Cat# 15596026
RIPA Lysis buffer (10x)	Merck Millipore	Cat # 20-188
PhosSTOP™ phosphatase inhibitor	Merck Roche	Cat # 4906837001
EASYpack™ Protease Inhibitor Cocktail	Merck Roche	Cat # 5892970001
Phenylmethylsulphonyl Fluoride (PMSF)	Life Technologies	Cat # 36978
Laemmli (SDS-Sample) Buffer (Reducing, 6X)	Enzo Life Sciences	Cat # NOV-NBP- 111R
Sodium dodecyl sulfate (SDS)	Sigma-Aldrich	Cat #L3771
Sodium Butyrate	Abcam	Cat # ab120948
DNase/RNase-Free Distilled Water	Life Technologies	Cat # 10977035
LightCycler 480 SYBR Green I Master Mix	Roche	Cat # 04707516001
EGF (Epidermal Growth Factor)	Thermo Fisher Scientific	Cat # PHG0313
FGF (Fibroblast Growth Factor )	Bio-Techne R&D	Cat # 233-FB- 025/CF
B27 Supplement	Thermo Fisher Scientific	Cat # 17504044
Low Melting Temperature Agarose	Sigma-Aldrich	Cat # A4018
UltraPure™ Agarose	Invitrogen	Cat # 16500500
Restriction digestion Enzyme BbsI-HF	NEB	Cat #R3539
Restriction digestion Enzyme KpnI-HF	NEB	Cat #R3142
Restriction digestion Enzyme BsiWI-HF	NEB	Cat #R3553
CutSmart™ Buffer	NEB	Cat #M0202M
T4 DNA ligase	NEB	Cat #M0202M
T4 DNA Ligase Reaction Buffer	NEB	Cat #B0202
Monarch RNase A (20 mg/mL)	NEB	Cat #T3018
Opti-MEM™ Reduced Serum Medium	Gibco	Cat # 31985062
DMEM High Glucose	Gibco	Cat # 11965092
RPMI 1640 Medium	Gibco	Cat # 61870036
RPMI 1640 with HEPES	Gibco	Cat # 22400089
DMEM, no glucose, no glutamine, no phenol red	Gibco	Cat # A1443001
Tumor Dissociation Kit, human	Miltenyi Biotec	Cat # 130-095-929
Red blood cell lysis buffer	BioLegend	Cat # 420301
Trypsin-EDTA (10x)	Gibco	Cat # 15400054
L-Glutamine (100x)	Gibco	Cat # 25030081
Fetal Bovine Serum	Gibco	Cat # 26140079
Penicillin/Streptomycin	Gibco	Cat # 10378016
Lipofectamine 2000 Transfection Reagent	Life Technologies	Cat # 11668019
Lipofectamine™ RNAiMAX Transfection Reagent	Invitrogen	Cat # 13778075
Polybrene Infection/Transfection Reagent	Merck Millipore	Cat # TR-1003-G
Puromycin	Life Technologies	Cat # A1113803
Bovine Serum Albumin	Merck Life Sciences	Cat # A9647
16% Pierce™ Methanol-free Formaldehyde	Thermo Fisher Scientific	Cat # 11586711
Tris Base	Merck Millipore	Cat # 648310-M
Dithiothreitol (DTT), reducing reagent	Abcam	Cat # ab141390
Sucrose	Merck Millipore	Cat #S0389
NP-40	Sigma-Aldrich	Cat #I8896
Trichloroacetic acid (TCA)	VWR	Cat # 89500-550
Trifluoracetic acid (TFA)	VWR	Cat # 153112E
Acetonitrile (ACN)	VWR	Cat # 83640.320
LC-MS Grade Water	Supelco	Cat # 115333.2500
Monarch® RNase A	NEB	Cat #T3018L
Proteinase K (20 mg/mL)	Thermo Fisher Scientific	Cat # EO0491
Vectashield™ Vibrance Antifade Mounting Medium	2BScientific	Cat # H-1700-10
D-Glucose (13C-6)	Merck Life Sciences	Cat # 389374
Sodium Acetate (13C-2)	CK Isotopes	Cat # CLM-381-1
Sodium Pyruvate (13C-3)	CK Isotopes	Cat # CLM-2440
FACSmax™Cell Dissociation Solution	AMS Biotechnology	Cat #T200100
Liberase TM Research Grade	Merck Roche	Cat # 5401119001
CPI-613 (Devimistat)	Axon Medchem	Cas # 95809-78-2
AC-148	Chan et al.^77^	N/A
vemurafenib (PLX4032)	SelleckChem	Cat #S1267
Nifuroxazide (NAZ)	Merck Millipore	Cat # 481984
Dimethyl sulfoxide (DMSO)	Sigma Aldrich	Cat # 67-68-5

**Critical commercial assays**		

Aldefluor Assay (with DEAB and 2N HCl included)	STEMCELL technology	Cat# 01705
AldeRed ALDH Detection Assay	Millipore Europe	Cat # SCR150
SureBeads™ Starter Kit Protein A and G	Bio-Rad	Cat #1614833
Direct-zol RNA Miniprep Kits	ZYMO Research	Cat# 2052
SuperScript III Reverse Transcriptase	Life Technologies	Cat # 18080044
Illumina TruSeq Stranded mRNA Sample Prep Kit	Illumina	Cat # RS-122-2001
NEBNext Ultra Directional RNA library Prep Kit	NEB	Cat #E7420S
NEBNext Ultra II DNA Library Prep Kit for Illumina	NEB	Cat #E7645S
NEBNext Multiplex Oligos for Illumina® (Index Primers Set 1)	NEB	Cat #E7335S
NEBNext High-Fidelity 2X PCR Master Mix	NEB	Cat #M0541S
AMPure XP beads	Beckman Coulter	Cat # A63882
QIAquick PCR Purification Kit	Qiagen	Cat # 28104
Pierce™ BCA Protein Assay Kit	Thermo Fisher	Cat # 23225
4–15% Mini-PROTEANTM TGX Precast Protein Gels	Bio-rad	Cat # 4561085DC
Trans-Blot™ Turbo Mini 0.2 μm Nitrocellulose Transfer Packs	Bio-rad	Cat # 1704158
Collagen I, Coverslip Glass 22mm Round	VWR	Cat # 734-1009
L-Lactate Assay Kit (Colorimetric/Fluorometric)	Abcam	Cat # ab65330
Zenon™ Rabbit IgG labelling kits Alexa Fluor 488	Thermo Fisher	Cat #Z25302
Zenon™ Rabbit IgG labelling kits Alexa Fluor 647	Thermo Fisher	Cat #Z25308
CUTANA™ pAG-Tn5 for CUT&Tag	EpiCypher	Cat # SKU: 15-1017
Magnetic Concanavalin A Beads	Bangs Laboratories	Cat # BP531
Spermidine 0.1 M solution	Sigma Aldrich	Cat # 05292-1ML-F
SureBeads™ magnetic protein A beads for co-immunoprecipitation	BioRad	Cat # 1614013

**Deposited data**		

Raw and analyzed A375 ALDH^High^ and ALDH^Low^ RNA-seq data	This paper	GEO: GSE243840
Raw and analyzed A375 Cas9 ALDH1A3 WT and ALDH1A3 KO ChIP-Histone acetyl-H3-seq data; CUT&TAG H3K23ac and H3K27ac data	This paper	GEO: GSE243834
AlphaFold multimer modeling result of ALDH1A3-ACSS2 interaction	This paper	ModelArchive: ma-i8503
AlphaFold multimer modeling result of Ald6-Acs2 interaction	This paper	Model Archive: ma-n4imc
Mouse melanoma single-cell RNAseq	Karras et al.^46^	GEO: GSE207592
Human patient melanoma single-cell RNAseq before immune checkpoint blockade therapy	Pozniak et al.^48^	EGA: EGAD00001009291
Patient melanoma single-cell RNAseq	Tirosh et al.^47^	GEO: GSE72056
Human cutaneous melanoma TCGA dataset	TCGA cBioPortal; https://www.cbioportal.org/	RRID: SCR_003193
Human cutaneous melanoma Lund dataset	Cirenajwis et al.^53^	GEO: GSE65904
Human cutaneous melanoma Bergen dataset	Jonsson et al.^54^	GEO: GSE22155
Tfap2b (biotin) ChIP-seq	Ling and Sauka-Spengler^105^	GEO: GSE125711
HA-MITF ChIP-seq	Louphrasitthiphol et al.^102^ Louphrasitthiphol et al.^103^	GEO: GSE137522; GSE137776

**Experimental models: Cell lines**		

Human: A375 melanoma cell line	ATCC	RRID: CVCL_6233
Human: Cas9 A375 melanoma cell line	David Adams, Sanger Institute, UK	N/A
Human: Cas9 C089 melanoma cell line	David Adams, Sanger Institute, UK	N/A
HEK293T	ATCC	Cat# CRL-3216; RRID: CVCL_0063
MPD001	This paper	N/A
MPD002	This paper	N/A

**Experimental models: Organisms/strains**		

Zebrafish: *Tg(mitfa:GFP, mitfa:BRAFV600E), p53*^*M214K*^	Patton et al.^79^	ZFIN Cat #: ZDB-TGCONSTRCT-070117-106, ZDB-ALT-050428-2

**Oligonucleotides** (See also Table S6)		

gALDH1A3: CGTCCCGGAGCAATCTGAAG	Sanjana et al.^106^	https://www.genscript.com/tools/gRNA-library-design
gTFAP2B: GGACCGAGTGGTAGTCCCTC	Sanjana et al.^106^	https://www.genscript.com/tools/gRNA-library-design
siALDH1A3: GUAUCGAAGAAGUGAUAAA	Life technology: Silencer® Select	Cat # 4390824

**Recombinant DNA**		

pKLV2-U6gRNA5(BbsI)-PGKpuro2A-BFP	Addgene	Cat # 67991
pKLV2-U6gRNA5(BbsI)-PGKpuro2A-mCherry	This paper	N/A
pKLV2-U6gALDH1A3-PGKpuro2A-BFP	This paper	N/A
pKLV2-U6gTFAP2B-PGKpuro2A-BFP	This paper	N/A
pKLV2-U6gRNA5(BbsI)-PGKpuro2A-ALDH1A3	This paper	N/A
pKLV2-U6gRNA5(BbsI)-PGKpuro2A-TFAP2B	This paper	N/A
psPAX2	Addgene	Cat # 12260
pMD2.G	Addgene	Cat # 12259

**Software and algorithms**		

R (v. 4.0.4)	R core team (2020): (https://www.R-project.org/.)	RRID:SCR_001905
R Studio (v. 1.4.1106)	RStudio Team (2020): (http://www.rstudio.com/.)	RRID:SCR_000432
ggPlot2 (v.3.4.2)	(Wickham, 2016) https://cran.r-project.org/web/packages/ggplot2/index.html	RRID:SCR_014601
MATLAB	The Math Works (2020): (http://www.mathworks.com/products/matlab/)	RRID:SCR_001622
FastQC (v.0.11.3)	Babraham Institute; (https://www.bioinformatics.babraham.ac.uk/projects/fastqc/)	RRID: SCR_014583
STAR (v. STAR_2.5.1b)	Dobin et al.^107^	RRID: SCR_015899
RNASeqQC (v.1.1.8.1)	DeLuca et al.^108^	RRID: SCR_005120
htseq-count (0.6.1)	Simon et al.^70^	RRID:SCR_011867
DESeq2 R package (v. 1.30.1)	Love et al.^70^	RRID: SCR_015687
GSEA software (v. 4.0.3)	Subramanian et al.^109^ (http://www.broad.mit.edu/gsea/index.html)	RRID: SCR_016884
g:Profiler	Raudvere et al.^64^	RRID:SCR_006809
Seurat (v. 4.0.0)	Hao et al.^110^	RRID:SCR_016341
EpiProfile2.1_1Basic	Yuan et al.^111^	N/A
Bowtie2 (bwa-0.7.9)	Langmead and Salzberg^70^	RRID:SCR_016368
HOMER (version 4.11)	Heinz et al.^112^	RRID:SCR_010881
ChIPseeker R package (v. 1.26.2)	Wang et al.^113^	RRID:SCR_021322
SAMtools (v. 1.9)	Li et al.^114^	RRID:SCR_002105
MACS2 (v. 2.1.1.20160309)	Feng et al.^115^	N/A
deeptools (v. 3.5.1)	Ramírez et al.^116^	RRID:SCR_016366
DiffBind (v. 2.10.0)	Ross-Innes et al.^117^	RRID:SCR_012918
edgeR (v3.32.1)	McCarthy et al.^118^	RRID:SCR_012802
GSVA (v1.38.2)	Hänzelmann et al.^119^	RRID:SCR_021058
Galaxy (usegalaxy.org)	The Galaxy Community^120^	RRID:SCR_006281
The UniProt Consortium	The UniProt Consortium^121^	RRID:SCR_002380
AlphaFold-Multimer	Evans et al.^76^	N/A
ColabFold version 1.5.2	Mirdita et al.^122^	N/A
GalaxyRefineComplex	Heo et al.^123^	N/A
FreeSASA 2.0.3	Mitternacht^124^	N/A
AlphaFill	Hekkelman et al.^125^	N/A
UCSF ChimeraX version 1.6	Pettersen et al.^126^	RRID:SCR_015872
ImageJ Fiji software (v. 1.53c)	National Institutes of Health, USA	RRID: SCR_003070
Prism 9 (v. 9.3.1) for macOS	GraphPad Software, San Diego, USA	RRID: SCR_002798
eulerr R (v. 6.1.1)	https://CRAN.R-project.org/package=eulerr	RRID:SCR_022753
Cytoscape (v.3.8.0)	Otasek et al.^127^	RRID:SCR_003032
SCENIC	Aibar et al.^70^	RRID:SCR_017247
AUCell	Aibar et al.^70^	RRID:SCR_021327
CytoTRACE	Gulati et al.^45^	RRID:SCR_022828
pheatmap	https://CRAN.R-project.org/package=pheatmap	RRID:SCR_016418
NDP.view 2 (v. 2.9.29)	Hamamatsu.com: NDP.view2 U12388-01 (http://www.hamamatsu.com/jp/en/U12388 −01.html)	N/A
FlowJo (v. 10.8.1) for macOS	Becton, Dickinson and Company (https://www.flowjo.com/)	RRID:SCR_008520

### Resource availability

#### Lead contact

Further information and requests for resources and reagents should be directed to and will be fulfilled by the lead contact, E. Elizabeth Patton (e.patton@ed.ac.uk).

#### Materials availability

Plasmids generated in this study will be made available upon request made to the lead contact.

Patient derived low passage melanoma cells are available, upon MTA approval, upon request to v.pavet@beatson.gla.ac.uk and o.sansom@beatson.gla.ac.uk.

#### Data and code availability

The RNA-seq, ChIP-seq, and CUTandTAG data generated in this study have been deposited at GEO and are publicly available as of the date of publication. Accession numbers are listed in the key resources table. The PDB files of ACSS2-ALDH1A3 and Acs2-Ald6 from AlphaFold-Multimer modeling are released on ModelArchive and are publicly available as of the date of publication with the DOI listed in the key resources table. No original code had been generated for this paper. Any additional information required to reanalyse the data reported in this paper is available from the lead contact upon request.

### Experimental models and study participant details

#### Zebrafish maintenance and husbandry

Zebrafish were maintained in accordance with UK Home Office regulations, UK Animals (Scientific Procedures) Act 1986, under project license P8F7F7E52. All experiments were approved by the Home Office and AWERB (University of Edinburgh Ethics Committee).

#### Zebrafish melanoma models

Zebrafish were genotyped using DNA extracted from tail fin clipped tissue by PCR to confirm the mutant allele status of *tp53*^*M214K*^ (referred to as *p53*^*−/−*^ *or p53 mutant*) and *mitfa:BRAF*^*V600E*^ as described in our previous publications.^79^ The emergence of melanoma is usually observed in individuals aged 3- to 6-month-old. Individuals used in this study for DMSO control versus vemurafenib or Nifuroxazide drug pellets treatment were aged 5- to 6-month-old when entering the treatment scheme. Both female and male individuals were admitted into the treatment course regardless of the sex.

#### Human melanoma cell line culture

A375 cells were cultured in DMEM high glucose media, C089 cells were cultured in RPMI 1640 media, and patient sample derived cell line MPD001 were cultured in RPMI 1640 with 25 mM HEPES. All media were supplemented with 2 mM L-glutamine and 10% fetal calf serum, and all cells grown at 37°C in a 5% CO_2_ humidified incubator. All cells have been routinely tested for mycoplasma, with the most recent test performed on June 21, 2023.

### Method details

#### Establishment and amplification of patient derived melanoma cell lines

Patients with melanoma were managed in accordance with the ethical principles of Declaration of Helsinki and in accordance with Good Clinical Practice as defined by the International Conference on Harmonisation. All patients gave informed written consent to participate in clinical trials or EAP or EAMS. Patient samples were collected with written full-informed consent under Manchester Cancer Research Center (MCRC) Biobank ethics application #07/H1003/161 + 5 and approval for the work under MCRC Biobank Access Committee application 13_RIMA_01. Tissue samples were collected from patients with cutaneous melanoma at The Christie NHS Foundation Trust. For cell line MPD001 and MPD002, tumor fragments from the lymphatic melanoma and skin lesion to chest wall metastases were obtained respectively during the surgical procedure and dissociation was performed on the same day utilising the human tumor dissociation kit (Miltenyi Biotec) following manufacturer’s instructions. Briefly, after mechanical dissociation with a scalpel, tissue was resuspended in RPMI media with a mixture of Kit Enzymes (H, R and A) and placed on the gentleMACS Dissociator, program 37°C _h_TDK_1 for 30 min. Next, cells were transferred to a 50 mL falcon tube and centrifuged at 300xg for 7 min, then resuspended and passed through a 70 μm cell strainer. This was centrifuged again (300xg for 7 min) and resuspended in 1X red blood cell lysis buffer (BioLegend) in deionized water, then incubated at room temperature, protected from light, for 15 min. After this step, the sample was centrifuged and finally resuspended and plated in a 10 cm cell culture Petri dish in RMPI medium supplemented with FBS (Gibco) (10%) and Penicillin/Streptomycin (Gibco) (1%). Cells were cultured in an incubator at 37°C with 5% CO_2._ Cell lines were frozen and stored after a 2-week amplification period, freezing performed using FBS with DMSO (10%). Cryotubes were placed in an isopropanol freezing container (Nalgene Cat. C1562-1EA) at −80°C for 24h for gentle freezing and transferred to liquid nitrogen tanks for long term storage.

#### Generation of human melanoma mutant cell lines

Human melanoma cell lines A375 and C089 engineered with stable expression of *Streptococcus pyogenes* Cas9 were gifted to us and used to build *ALDH1A3* mutant cells.^42^ Briefly, for *ALDH1A3* knockout, the vehicle plasmid expressing gRNA (pKLV2-U6gRNA5(BbsI)-PGKpuro2ABFP, Addgene: 67991) was engineered by Golden Gate cloning using restriction enzyme BbsI-HF (NEB) to express gRNA targeting ALDH1A3 (CGTCCCGGAGCAATCTGAAG). Lentiviral particles were produced by co-transforming the HEK293T (ATCC) cells with target plasmid, the packaging plasmid psPAX2 (Addgene: 12260), and pMD2.G (Addgene: 12259), facilitated with Lipofectamine 2000 (Invitrogen) following the manufacturer’s instructions. 48 h post transfection, the 293T cell culture supernatant was collected and filtered (0.45 μm) to transfect the targeted recipient cell lines, supplemented with 10 μg/mL polybrene (Merck Millipore). 48–72 h post transfection, the cells were split and seeded with the complete growth media containing 1 μg/mL puromycin (Life Technologies) to obtain clones with stable expression of the gRNA. Suitable single clones were validated by western blot to confirm full-length *ALDH1A3* knockout and Aldefluor assay to confirm the loss of ALDH activity before expanded for biological experiments. For *TFAP2B* knockout cells, the gRNA sequence was engineered similarly (GGACCGAGTGGTAGTCCCTC) using Golden Gate cloning. For vehicle control samples, the vehicle plasmid expressing empty gRNA were engineered to express mCherry instead of *BFP* sequence; for *ALDH1A3* and *TFAP2B* over-expression cells, the vehicle plasmid expressing empty gRNA were engineered to express *ALDH1A3* CDS instead of *BFP*. Briefly, restriction digestion enzyme KpnI-HF (NEB) and BsiWI-HF (NEB) were used to remove *BFP* sequence and create sticky ends matching the *mCherry*, *ALDH1A3* or *TFAP2B* CDS flanking sequence. The digested fragment of plasmid backbone and the target sequence were then ligated using T4 DNA ligase (NEB), following which a similar transfection and selection procedures were performed to acquire stable mutant lines.

#### Human melanoma cell ALDH activity measurement

The ALDH enzyme activity in human melanoma cells was measured using the Aldefluor assay kit (StemCell Technologies) following the manufacturer’s instructions. In brief, melanoma cells dissociated with trypsin (Gibco) were resuspended in the Aldefluor buffer with the fluorescent bodipy-aminoacetaldehyde (BAAA) reagent included in the kits. For each experiment, a negative control vial was set up using a small aliquot out of the sample vial (100 μL out of 1 mL), supplemented with (5 μL) pan-ALDH inhibitor DEAB (diemethylaminobenzaldehyde) immediately after the resuspension. After incubation of 30 min at 37°C, the Aldefluor activity was measured using flow cytometry (Fortessa, BD Biosciences). For cell sorting to establish the ALDH^High^ and ALDH^Low^ cells, the stained cells were sorted by FACS Aria II (BD Biosciences) and the population with the highest and lowest 5% ALDH activity (ALDH^High^ and ALDH^Low^) were collected. All flow cytometry data were analyzed using the software FlowJo. Dead cells were excluded using 7-Aminoactinomycin D (7-AAD, Eurogentec), or 4,6-Diamidine-2-phenylindole dihydrochloride (DAPI, Sigma-Aldrich).

#### RNA extraction and RT-qPCR

Quantitative reverse transcription PCR (RT-qPCR) assays were performed by standard protocol suitable for LightCycler 480 Instrument (Roche). In brief, total RNA was extracted and purified from live cells using TRIzol (Invitrogen) and Direct-Zol RNA Miniprep Kits (ZYMO Research). After quality check and measurement by NanoDrop (ThermoFisher), 1 μg RNA for each sample was reverse transcribed using Superscript III reverse transcriptase (Life Tchnologies). Quantitative PCR were carried out by setting up reactions using the reverse transcribed cDNA template, primers (see also Table S6, related to STAR Oligonucleotides) and LightCycler 480 SYBR Green I Master reagent, run by the program of Standard Roche Template (System II). Reads of gene ACTB (Beta Actin) were used as the internal control to calculate the relative expression values.

#### RNA-seq pipeline

Libraries were prepared from 500 ng of each total-RNA sample using the TruSeq Stranded mRNA Library Kit (Illumina) according to the provided protocol and purified using AMPure XP beads (Beckman Coulter). Sequencing was performed using the NextSeq 500/550 High-Output v2 (150 cycle) Kit (# FC-404-2002) on the NextSeq 550 platform (Illumina Inc, #SY-415-1002). Libraries were combined in an equimolar pool based on the library quantification results and run across a single High-Output Flow Cell. Raw FASTQ sequence reads were quality checked using FastQC (v. 0.11.3) and aligned to the human genome (GrCh38) assembly using STAR (v. STAR_2.5.1b) software with default parameters. The quality of the resulting alignment to the transcriptome (Ensembl annotation version GRCh38.91) was checked using RNASeqQC (v. 1.1.8.1). Raw counts of reads covering the transcriptome (Ensembl annotation version GRCh38.91) were obtained using htseq-count (0.6.1) with the “-s reverse” option. Differential expression was analyzed using the DESeq2 R package (v. 1.20.0).

#### Pathway enrichment analysis

Gene set functional enrichment analysis (GSEA) identified enriched pathways at FDR <0.05 (weighted Kolmogorov-Smirnov test) using GSEA software (Subramanian et al., 2005). The gene expression matrix of sorted ALDH^High^ versus ALDH^Low^ cells or selected patient melanoma ALDH1A3^High^ versus ALDH1A3^Low^ samples were used to compare against literature-based datasets (Tables S1 and S2) using gene set permutation settings. For gene over-representation analysis across literature curated signature terms, genes with differential acetyl-Histone H3 in WT compared to *ALDH1A3* knockout were selected based on FDR <0.05 (in total 1599, see also Table S4) as input to the g:Profiler website.^64^ The gene terms with FDR <0.05 (hypergeometric test, BH adjusted) were considered significantly over-represented, with the term size cut-off set to 1000 (token: gp__33Kp_FaEr_Mcs). For gene over-representation analysis across the REACTOME database,^128^ genes with acetyl-histone H3 peaks differentially enriched in WT or *ALDH1A3* knockout (see also Table S4) were separately tested and terms of FDR <0.05 (hypergeometric test, BH adjusted) were considered significantly over-represented.

#### Gene set expression correlation analysis

To assess the expression corelation between *ALDH1A3* and NCSC gene signature, gene set variation analysis (GSVA)^118^ was used to calculate the relative expression score for the gene set containing only ALDH1A3 or the related signature gene lists. Spearman correlation analysis was then performed to evaluate the statistical correlation across all samples. Positive correlation was determined as Spearman co-efficient R > 0.3 with exact critical probability p-value <0.05.

#### Human metastatic melanoma single cell data mining

Human metastatic melanoma single-cell RNA-seq expression matrix was accessed via supplementary data of Tirosh et al., 2016.^47^ Cells classified as malignant tumor cells were extracted for UMAP clustering via Seurat (v.4.0.0), with *ALDH1A3* and *TFAP2B* visualised, clustering resolutions equals 0.5. For immune immune checkpoint blockade therapy naive samples, single-cell RNA-seq expression matrix of human melanoma was accessed via the published study of Pozniak et al., 2024^48^ as deposited on European Genome-phenome Archive (EGA): EGAD00001009291, with the original patient tumor progression tracked and recorded for their anti-PD-1 and/or anti-CTLA-4 treatment outcomes. Cells classified as malignant tumor cells were extracted for violin plot analysis via Seurat (v.4.0.0), with *ALDH1A3* visualised and compared between the responder and non-responder groups based on the matched patient clinical records.

#### *NRAS*^*Q61K/°*^*; Ink4a*^*−/−*^ murine melanoma data mining

Single-cell RNA expression data of malignant melanoma cells, originating from 5 primary murine melanoma lesions,^46^ were interrogated for *Aldh1a3*, ALDHhigh_enriched_signature and Tfap2b_regulon expression activities, measured by AUCell.^70^ A Tfap2b regulon is inferred by using the pySCENIC pipeline on a mouse melanoma single-cell RNA-seq data^46^ with the genes extracted and visualized using Cytoscape (v.3.8.0).^129^ To map potential differentiation trajectories onto the single-cell UMAP space, we calculated for each melanoma cell its corresponding CytoTRACE score,^45^ which is a measure of gene expression diversity and a surrogate for developmental potential (0<CytoTRACE score<1). CytoTRACE scores close to 1 are indicative of a less differentiated and close to 0 of a differentiated state. Besides the single cells, every gene was scored and correlated to either contribute to a dedifferentiated or differentiated state and ranked accordingly.

#### Western blot

Cells were detached by Trypsin (Gibco) and lysed on ice for 30 min at a cell density of 10^7^ cells per mL of RIPA lysis buffer (Merck Millipore), supplemented with phosphatase and complete protease inhibitors (Merck Roche). Debris of cells were removed by centrifuge (10,000 rpm, 10 min, 4°C). Protein concentrations were determined using the BCA assay (Thermo Fisher) and 10–20 μg protein per lane was electrophoresed on 4–15% precast gradient gels (Bio-Rad). Based on the protein concentration and sample volumes, calculated amount of Laemmli (SDS) buffer (Enzo Life Science) were added and incubated with the samples at 95°C for 5 min before gel loading. After the electrophorese program, gels were transferred onto Turbo transfer membranes (Bio-Rad) using the semi-dry Turbo Transfer system (Bio-Rad). Membranes were then blocked with 5% w/v BSA/TBS for 30 min at room temperature, subsequently probed by primary antibodies with optimised dilution factors overnight at 4°C, incubation of goat anti-mouse IRDye 680- or goat anti-rabbit 800- labelled secondary antibodies (LI-COR Biosciences) and imaged using an Odyssey infrared scanner (LI-COR Biosciences).

To probe the histone proteins using western blot, an acid extraction protocol was carried out instead of RIPA lysing protocol. In brief, the cells were washed with ice-cold PBS and lysed on ice for 10 min at a cell density of 10^7^ cells per mL of Triton Extraction Buffer (TEB: PBS, 0.5% Triton X-100 (v/v), 2 mM phenylmethylsulfonyl fluoride (PMSF, Life Technologies)), supplemented with 5 mM sodium butyrate (Abcam) to retain levels of histone acetylation. The nuclei of cells were centrifuged (650 x g, 10 min, 4°C) and washed in half the volume of TEB and centrifuged again. Pellets were re-suspended using 0.2 N HCl at a density of 4x10^7^ nuclei per mL. The histones were extracted overnight at 4°C and the debris were removed by centrifuge (650 x g, 10 min, 4°C). 5M NaOH were added at 1/20 of the volume of the supernatant to neutralise the samples. The same steps of protein measurement and western blot were followed as described above for probing protein in the total cell lysates.

The primary antibodies used are as following: ALDH1A3 (1:10,000, Rabbit, Invitrogen), TFAP2B (1:1000, Mouse, Santa Cruz), beta-Actin (1:1000, Mouse, Invitrogen), Histone H3 (1:1000, Mouse, Abcam), acetyl-Histone H3 (1:10,000, Rabbit, Millipore).

#### Co-immunoprecipitation of ACSS2

Co-immunoprecipitation was performed using SureBeads Protein A Magnetic Beads (BioRad) following recommended instructions from the manufacturer. Briefly, to prepare the beads for binding target protein, for each 1 million cells, 5 μL SureBeads were washed in 0.1% TBST for 3 times and then incubated with 1:50 ACSS2 primary antibody (Rabbit, Cell Signaling) or 1:50 IgG control (Cell Signaling Technology) at room temperature for 30 min. Unbound antibodies were removed by another 3 times of washing using 0.1% TBST. To prepare the protein lysates, cells were cultured to 80–90% confluence before dissociated using trypsin/EDTA. Cells were pooled into a master tube before aliquoting 20 million cells per sample for whole cell co-IP or 40 million cells per sample for nuclear department co-IP.

For whole-cell co-IP, aliquoted cells were pelleted by centrifuge at 300 xg, for 3 min and resuspended in 1x RIPA buffer supplemented with Roche protease inhibitor cocktail and lysed on ice for 10 min. The lysates were then centrifuged at 12,000 xg for 10 min before the supernatant were transferred to primary antibody conjugated SureBeads Protein A Magnetic Beads for incubation overnight at 4°C. For nuclear protein co-IP, nuclei isolation were performed by adding 5 mL nuclei isolation buffer (NIB) to the cell pellets (15 mM Tris, 60 mM KCl, 15 mM NaCl, 5 mM MgCl_2_, 1 mM CaCl_2_, 250 mM sucrose, pH adjusted to 7.5, supplemented with 1 mM DTT and 0.1% NP-40) with gentle pipetting and incubation on ice for 5 min. Nuclei were spun down by centrifuging at 600 xg for 5 min at 4°C and then proceed to the same RIPA lysing steps as described for the whole cell co-IP assay. For both whole-cell and nuclei co-IP, 10% of lysates were set aside as input before proceeding to adding SureBeads conjugated antibodies.

Following incubation at 4°C overnight, unbound proteins were washed off by rinsing the magnetic beads 3 times using 0.1% TBST. Captured proteins were released by adding 50 μL 1x RIPA buffer and 10 μL 6x Laemmli SDS reducing buffer to the beads slurry and being heated at 95°C for 3 min. Proteins were then probed by standard Western Blot as described above.

#### Colony formation assay in soft agar

For each sample in a 6-well plate set up, 5000 cells were suspended in serum-free growth media supplemented with 10 ng/mL EGF, 10 ng/mL FGF, B27 supplement (1x, Thermo Fisher Scientific), and 0.3% low melting agarose (Sigma-Aldrich). Cells were then layered over a solid base of 0.5% low melting agarose, cultured for 18 days (A375) or 25 days (C089), optimised by the colony growth speed. Colonies (>10 cells) from 10 separate fields of each sample were then manually counted using the images captured by a Nikon DS-L3 camera system on an Eclipse TS100 microscope (Nikon) with a 4× objective.

#### Immunocytochemistry with fluorescence labelling and imaging

Cells were cultured on the collagen I coated coverslip glasses (VWR) for ICC assays. Samples were washed twice with PBS and fixed with 4% paraformaldehyde (PFA, Thermo Fisher Scientific) at room temperature for 15 min before permeabilized by PBST (0.1% Triton x-100/PBS) for 10 min. Coverslips were then incubated in 3% BSA/PBST (w/v) for 30 min before probed with primary antibodies at appropriate dilution for 4 h at room temperature or 4°C overnight (ALDH1A3, 1:300, Abcam; TFAP2B, 1:100; ACSS2, 1:100, Abcam). After being washed 3 times in PBST for 5 min each, samples were incubated with fluorochrome-conjugated secondary antibodies (Donkey anti-Rabbit 488, Donkey anti-Mouse 568, and Donkey anti-Rabbit 647, all 1:1000, Invitrogen) for 30 min in the dark. After 3 times of wash in PBST for 5 min each, DAPI (Sigma-Aldrich) was added to the final wash of PBST to stain the nucleus, and the coverslips were mounted with antifade mounting medium (Vectashield, 2BScientific) before fluorescent microscope imaging using multimodal Imaging Platform Dragonfly (Andor technologies, Belfast UK). Images were acquired using a 20X or 40X lens equipped with 405, 488, 561, and 640 nm lasers built on a Nikon Eclipse Ti-E inverted microscope body with Perfect focus system (Nikon Instruments, Japan). Data were collected in Spinning Disk 25 μm pinhole mode on the Zyla 4.2 sCMOS camera using a Bin of 1 and no frame averaging using Andor Fusion acquisition software.

For ICC on flow cytometry sorted ALDH^High^ and ALDH^Low^ cells, Aldefluor-sorted cells were resuspended in normal culture media and allow to attach on the collagen I coated coverslip glasses (VWR) in 6-well plates for 4–6 h before fixed to ensure the ALDH activity states.

For interrogating ALDH1A3-TFAP2B-ACSS2 co-localisation, primary antibodies raised in the same species, *i.e.* anti-ALDH1A3 and anti-ACSS2 Rabbit IgG, were pre-conjugated to different fluorescence dye (Alexa Fluor 488 and Alexa Fluor 647) separately following the manufacturer’s instructions (Zenon Rabbit IgG labelling kits, Thermo Fisher) before incubation with the samples together with Mouse anti-TFAP2B primary antibody for 2 h at room temperature. The cells were then washed three times in PBS and incubated with Donkey-*anti*-mouse conjugated Alexa Fluor 568 antibodies for 0.5 h at room temperature (Invitrogen). Cells were washed, with nuclei stained with DAPI (Sigma) and mounted in Vectashield mounting media before imaging. Super-resolution images were acquired using instant Structured Illumination Microscopy (SIM)^130^ with Nikon SoRa system. Imaging was carried out using an SR HP Plan Apo λS 100x 1.35NA Silicone lens (Nikon Instruments). The CMOS cameras used for acquisition were Teledyne Photometrics Prime 95B (Teledyne Photometrics 3440 E.Britannia Drive, Tucson AZ) and 405/488/514/561/640nm laser lines. Z-step size for Z stacks was set to 0.120 μm as required by manufacturers software. Acquisition of images and deconvolution was carried out using the (3D algorithm) Nikon NIS Elements Advanced Research software. Settings for acquisition and reconstruction were identical in all images. Line scanning signal correlation analysis were carried out in Fiji by taking the intensity from each channel across the scanning line as an individual variable and conducting Pearson correlation test.

#### ^13^C_6_-glucose tracing via targeted UPLC-MRM/MS

For ^13^C_6_-glucose tracing experiment, DMEM with no glucose, no glutamine, no phenol red (Gibco) was purchased and then supplemented with 4.5 g/L ^13^C_6_-glucose (Merck Life Sciences), 2 mM L-glutamine, and 10% FCS (referred to from now on as the ^13^C_6_-glucose DMEM). To trace ^13^C_6_-glucose in A375 ALDH^High^ and ALDH^Low^ cells, A375 melanoma cells were cultured in T175 flasks (Corning) until reaching 70% confluence, then incubated in ^13^C_6_-glucose DMEM for 12 h before dissociated for Aldefluor staining and live sorted by FACS Aria II (BD Biosciences). Every sample contains ∼0.5 million sorted cells, which was immediately snap frozen in liquid nitrogen upon sorting. To trace ^13^C_6_-glucose in A375 Cas9 control and *ALDH1A3* knockout cells, cells were seeded in 6-well plates until reaching 50% confluence, then incubated in ^13^C_6_-glucose DMEM for 24 h before trypsin dissociation and liquid nitrogen snap frozen. Metabolites extraction, derivation, and targeted UPLC-MRM/MS profiling the central carbon metabolites were performed as described in Han et al., 2013^131^ by the service at Metabolomics Innovation Center, University of Victoria.

#### Histone acetylation profiling using bottom-up mass spectrometry

Histone extraction and derivatization workflow was optimised based on Sidoli et al., protocol.^132^ Briefly, nuclei from live attached melanoma cells were isolated by 5-min incubation on ice with NIB buffer (15 mM Tris, 60 mM KCl, 15 mM NaCl, 5 mM MgCl_2_, 1 mM CaCl_2_, 250 mM sucrose, pH adjusted to 7.5, supplemented with 1 mM DTT, 0.5 mM PMSF, 0.05 μM NaF, 0.05 μM NaVO_4_ and 10 mM sodium butyrate, 0.1% NP-40) followed by scraping. Nuclei were spin down by centrifuging at 1000 rcf for 5 min at 4°C. The nuclei pellets were washed twice by NIB without NP-40. Histone proteins were extracted by adding 0.2 M H_2_SO_4_ in a 1:4 ratio of nuclear pellet to H_2_SO_4_ and incubated for 4 h at 4°C. Histones were then precipitated by adding 1:3 v/v Trichloroacetic acid (TCA) and incubated overnight at 4°C. For chemical derivatization, precipitated histone proteins were air-dried by vacuum centrifuge and rinsed with ice-cold acetone prior to four rounds of propionylation, with the last two rounds of propionylation carried out on histone peptides post trypsin digestion. Propionlated peptides were transferred to C18 staging tips for desalting and eluted using 80% acetonitrile (ACN) with 0.1% trifluoracetic acid (TFA) before LC-MS analysis.

Peptides resulting from all digestions were separated by nanoscale C18 reverse-phase liquid chromatography using an UltiMate 3000 RSLCnano system coupled online to an Orbitrap Fusion Lumos Tribrid mass spectrometer (Lumos) (all Thermo Fisher Scientific). HPLC buffers- 0.1% formic acid in HPLC-grade water (buffer A); 0.1% formic acid in HPLC-grade acetonitrile (buffer B) were prepared. HPLC method was programmed as follows: from 0 to 30% buffer B in 30 min, from 30 to 100% B for the next 5 min and at isocratic 100% B for 8 min the flow rate was set to 250–300 nL/min.

Acquisitions were carried out in data independent acquisition mode (DIA) using Tune application 3.5.3890 (Thermo Scientific). A nanoelectrospray ion source (Sonation) was used for ionisation in positive mode. Chromatography was carried out at a flow rate of 250–300 nL/min using 50 cm fused silica emitters (CoAnn Technologies) packed in house with reverse phase Reprosil Pur Basic 1.9 μm (Dr. Maisch GmbH). The emitter was heated to 50°C using a column oven (Sonation), and an Active Background Ion Reduction Device (ABIRD) was used to decrease air contaminants signal level. Peptides were eluted with a 60-min two-step gradient, over a total run time of 90 min. A full scan was acquired at a resolution of 60,000 at 200 m/z, over mass range of 300–1100 m/z, followed by a DIA scan. All precursors were fragmented using 15 consecutive windows with 50 Da width, allowing for a 1 m/z overlap, covering a mass range from 349.5 to 1100.5 m/z. Higher energy collisional dissociation fragmentation spectra were recorded at 15,000 resolution at 200 m/z. All ions were fragmented using normalised collision energy of 28%, for a maximum injection time of 54 ms, or a normalised AGC target of 1000%.

Data analysis was performed on MATLAB using the EpiProfile2.1_1Basic package (https://doi.org/10.1021/acs.jproteome.8b00133) using label-free settings (nsource = 1) or for histone H3 acetylation and 13C2-acetyl incorporation analysis the C13 on acetylation group (nsource = 3). Isotopic correction was performed by MATLAB as implemented in EpiProfile.

#### Acetyl-CoA extraction and LC-MS analysis

To extract acetyl-CoA from whole cell lysates and nuclear department, samples were collected as described by Trefely et al., protocol.^133^ Briefly, live attached melanoma cells were isolated by 5-min incubation on ice with NIB buffer (15 mM Tris, 60 mM KCl, 15 mM NaCl, 5 mM MgCl_2_, 1 mM CaCl_2_, 250 mM sucrose, pH adjusted to 7.5, supplemented with 1 mM DTT and 0.1% NP-40) followed by scraping. 10% of each sample were removed and quenched in 1 mL ice-cold 10% TCA as total lysates. Nuclei were spun down by centrifuging at 600 rcf for 5 min at 4°C. The nuclei pellets were washed twice by NIB without NP-40 and then quenched in 1 mL 10% TCA. Acetyl-CoA was extracted using solid phase extraction to remove TCA. Extraction cartridges (Oasis HLB 1cc (30 mg)) were conditioned by running 1000 μL of methanol (MeOH) following by 1000 μL of water (H_2_O). On the cartridges, 100 μL of samples were then loaded, washed with 1000 μL of H_2_O and eluted with 500 μL of MeOH. The extracts were then dried under nitrogen and reconstituted in 80% ACN: 20% H_2_O (20 mM ammonium carbonate 0.1% ammonium hydroxide solution 25%).

Samples were analyzed on a Dionex UltiMate 3000 LC System (Thermo Scientific, Waltham, Massachusetts, EUA) coupled to a Q Exactive Orbitrap Mass Spectrometer (Thermo Scientific, Waltham, Massachusetts, EUA) operating in negative polarity with scan range from 806 to 815 m/z. Chromatographic separation was achieved using a ZIC-pHILIC 150 × 2.1 mm column (Merck Millipore Sigma, Burlington, Massachusetts, EUA) at 45°C using a gradient starting from 20% buffer A (20 mM ammonium carbonate 0.1% ammonium hydroxide solution 25%), and 80% B (acetonitrile) to 80% buffer A, 20% buffer B at 9.5 min and reconditioning the column to the initial condition until 14.5 min. Mass spectrometry data were processed using Skyline^134^ on a targeted fashion by matching accurate mass and retention time with standard.

#### A375 acetyl-histone H3 ChIP-Seq

The control and *ALDH1A3* KO melanoma cells were cultured to 80% confluency and harvested by dissociation with trypsin in PBS/EDTA. Cells were resuspended in PBS and immediately fixed in 1% formaldehyde in PBS for 10 min at room temperature. The fixation was terminated by adding glycine and incubated for an additional 5 min. After being washed in cold PBS, cell pellets were resuspended in 150 μL chilled lysis buffer (1% SDS, 10mM EDTA, 50 mM Tris-HCl pH8.1, 1x protease inhibitor cocktail, 1x PhosSTOP phosphatase inhibitors (Roche), 5 mM sodium butyrate (Sigma) and fresh 1mM DTT) and supplemented with 850 μL 1% Triton X- IP dilution buffer (1% Triton X-, 20mM Tris-HCl pH8.1, 150mM NaCl, 2mM EDTA, 1x protease inhibitor cocktail (Roche), 1x PhosSTOP phosphatase inhibitors (Roche), 1mM DTT, 5 mM sodium butyrate and 1 mM PMSF) and incubated on ice for 10 min. Lysed cells were sonicated on ice for 8 × 30 s on/30 s off burst cycles with a probe sonicator (SoniPrep150) in a chilled ice-water bath (12 Amplitude) to yield chromatin fragments ranging between 200 and 800 bp in length. Sheared chromatin was centrifuged at 16,000xg for 10 min at 4°C and the soluble supernatant transferred to new tubes. Each 500 μL chromatin samples were supplemented with 5 μL (5 mg/ml) BSA. 10% of the input was stored and the rest was used for the immunoprecipitation.

Rabbit anti-acetyl-Histone H3 Antibody (Millipore) were pre-bound to magnetic SureBeads (BioRad) in 10% w/v BSA in PBS according to the manufacturer’s instructions for approximately 1h at 4°C with rotation, following which free antibody was removed with 3 washes of cold 10% w/v BSA in PBS. Chromatin and proteinG beads were combined and incubated over night at 4°C (at a ratio of 500 ng of bead bound antibody per 1 million cell equivalents of chromatin). Samples were washes at 4°C with rotation through the following series: 2 times in 1% Triton X- IP dilution buffer, 2 times with ChIP wash A (50mM HEPES ph7.9, 500mM NaCl, 1mM EDTA, 1% Triton X-100, 0.1% Na-deoxycholate, 0.1% SDS. 1x protease inhibitor cocktail, 1x PhosSTOP phosphatase inhibitors (Roche) and fresh 1mM DTT) and 2 times with ChIP wash B (20mM Tris pH 8.0, 1mM EDTA, 250mM LiCl, 1% NP-40, 0.1% Na-deoxycholate, 1x protease inhibitor cocktail, 1x PhosSTOP phosphatase inhibitors (Roche) and fresh 1mM DTT). Finally, the samples were washed with TE (1mM EDTA, 10mM Tris pH8.0). The samples were resuspended in TE and supplemented with preheated 37°C Extraction buffer (0.1M NaHCO3 and 1% SDS), vortexed and incubated for 15 min at 37°C on a vibrating platform.

The pH of the extracted chromatin was adjusted by adding 6μL 2M Tris-HCl pH6.8 following which both the ChIP and input samples were incubated with 20μg RNAse A (NEB) at 65°C for 1 h. Cross-links were reversed and the protein degraded by the addition of 20μg Proteinase K and incubation at 65°C for 6–8 h. Following removal of the magnetic SureBeads from the ChIP samples, DNA was purified using a Qiagen PCR cleanup kit following manufacturer’s instructions. DNA libraries were prepared using NEBNext Ultra II DNA Library Prep Kit for Illumina and NEBNext Multiplex Oligos for Illumina (Index Primers Set 1) following manufacturer’s instructions. Sequencing was performed using the NextSeq 500/550 High-Output v2.5 (150 cycle) Kit (#20024907) on the NextSeq 550 platform (Illumina Inc, #SY-415-1002). PhiX Control v3 (Illumina, #FC-110-3001) was spiked into the library pool at a concentration of ∼1% to enable troubleshooting in the event of any issues with the run.

#### ChIP-seq data analysis pipeline

Basecall data produced by the NextSeq 550 is automatically uploaded to BaseSpace, a cloud-based data management and analysis service provided by Illumina. Here it is converted into FASTQ files and mapped to was mapped to the human genome (GRCh19) using bowtie2 (bwa-0.7.9) applications directly accessible through BaseSpace. BAM files were uploaded to the open access bioinformatic community platform Galaxy (the public server at usegalaxy.org) for downstream analysis.^120^ All datasets and analysis history can be accessed (via https://usegalaxy.org/u/yuting_lu/h/a375-acetyl-histone-h3-chipseq-wt-vs-aldh1a3-knockout).

Briefly, mapped regions (due to fragment processing) extended beyond the end of the chromosomes were removed using SAMtools. MACS2 calling broadpeak algorithm was used to identify histone H3 acetylation regions, with each ChIP BAM file paired with the input control, cut off -- mfold 5 50, bandwidth --bw 500, FDR --qvalue 0.05. Call peak results (gapped peaks) were used for differential binding (histone acetylation in this case) analysis between A375 WT and ALDH1A3 knockout cells using the DiffBind.^117^ R package ChIPseeker^135^ was used for adjacent gene annotation and genomic distribution analysis. To visualise the histone H3 acetylation sites on the genome browser UCSC^136^ mapped reads were converted to bigWig files (.bw) using the bamCoverage (deepTools2) algorithm,^116^ with replicants for each condition merged.

#### ACSS2 ChIP qPCR

ACSS2 bound chromatin were pulled down using rabbit anti-ACSS2 antibody (Cell Signaling Technology) with the same ChIP protocol described above in A375 acetyl-Histone H3 ChIP-seq, ChIP-ACSS2 DNA and input control were purified using the QIAquick PCR Purification Kit (Qiagen) and used for qPCR with the same LightCycler 480 SYBR Green (Roche) system as described above in the qPCR method for RT-qPCR. The ChIP qPCR primer sequences are listed in Table S6.

#### MPD002 ALDH1A3 knockdown

ALDH1A3 knockdown was achieved using siRNA Transfection. In brief, MPD002 cells were seeded in 6-well plate at a density of 5 × 10^4^ cells/well and incubated overnight for attachment. Cells were then incubated with 10 nM ALDH1A3 siRNA or scrambled control (Life Technology) in 0.7 mL Opti-MEM media with Lipofectamine RNAiMAX (4.5μL/well)(Invitrogen) for 6 h. Medium was then changed to normal culture condition for another 42–64 h (48-72h in total post transfection) and the cells were collected for downstream analysis.

#### MPD002 acetyl-histone H3K23 and H3K27 CUT&TAG

The CUT&TAG experiments were performed following the protocol established by the Henikoff lab^137^ in principle. In brief, the control and *ALDH1A3* knockdown melanoma cells as well as spike-in control mouse embryonic stem cells (mESCs) were cultured to 90% confluency in 6-well plate and harvested by dissociation with trypsin in PBS/EDTA. Cells were resuspended in PBS supplemented with 5 mM sodium butyrate (Sigma) and counted. For each sample, 50,000 target cells (MPD002) and 5,000 spike-in control cells (mESCs) were combined and aliquoted for nuclei extraction (50 μL per sample, 20 mM HEPES pH7.5; 10 mM KCl; 0.5 mM Spermidine; 0.1% Triton; 1× Protease inhibitor cocktail; 20% v/v glycerol; 10 min on ice). Nuclei pellets were then collected by centrifuge for 4 min at 1,300 × g at 4°C and washed once in PBS supplemented with 5 mM sodium butyrate (Sigma) before binding to prepared concanavalin A coated magnetic beads (Bangs Laboratories, add 3.5 μl per sample to 50 μL binding buffer: 20 mM HEPES pH 8.0; 10 mM KCl; 1 mM CaCl2; 1 mM MnCl2; incubate 20 min at RT). The bead-bound nuclei were resuspended in 25 μL Wash Buffer (20 mM HEPES pH 7.5; 150 mM NaCl; 0.5 mM Spermidine; 1× Protease inhibitor cocktail) and the appropriate primary antibodies were added at 1:50 dilution ratio (Rabbit anti-H3K23ac, Invitorgen; Rabbit anti-H3K27ac, Cell Signaling Technology; CUT&TAG IgG control, Cell Signaling Technology). Primary antibody incubation was performed on a rotating platform overnight at 4 °C. Unbound primary antibodies were removed by placing the sample tubes to magnet stand and discarding all supernatant liquid. Next, 25 μL of 1:100 diluted goat anti-rabbit unconjugated secondary antibodies (Abcam) were added to each sample to increase the number of Protein A binding sites, with samples incubated on a rotating platform at RT for 1 h. Samples were then rinsed in Wash Buffer for 2–3 times before 1:200 dilution of pA-Tn5 adapter complex (EpiCypher) were prepared in high salt wash buffer (20 mM HEPES, pH 7.5, 300 mM NaCl, 0.5 mM Spermidine, 1× Protease inhibitor cocktail) and added to each sample (1.25 μl for each 25 μl reaction volume). pA-Tn5 incubation was performed at RT for 1 h on a rotating platform, before the unbound pA-Tn5 enzymes were removed by washing the beads using Wash Buffer on a magnet stand. Finally, tagmentation reaction were performed by resuspending the pA-Tn5 bound samples in 50 μL tagmentation buffer (High Salt Wash Buffer plus 10 mM MgCl2) and incubate at 37°C for 1 h. Tagmentation was terminated by removing the tagmentation buffer using magnet stand and wash once in TAPS wash buffer (10 mM TAPS, 0.2 mM EDTA). To release the DNA, 5 μL SDS release buffer was added to each sample (0.1% SDS in 10 mM TAPS, 0.2 mM EDTA) with samples incubated at 58°C for 1 h in a PCR cycler.

To prepare the libraries for sequencing, 15 μL of 0.67% Triton water solution were added directly to the bead slurry, plus 2.5 μL each of 10 μM uniquely barcoded i5 primer and i7 primers, using a different barcode combination for each sample. Next, 25 μL NEBNext HiFi 2× PCR Master mix (NEB) was immediately added and mixed before proceeding to PCR cycles (58 °C for 5 min (gap filling); 72 °C for 5 min (gap filling); 98 °C for 30 s; 14 cycles of 98 °C for 10 s and 60 °C for 10 s; final extension at 72 °C for 1 min and hold at 10 °C). Post-PCR clean-up was performed by adding 1.3× volume (65 μL) of Ampure XP beads (Beckman Counter) to the PCR reaction (50 μL) and incubating for 15 min at RT. The beads were then washed twice in 80% ethanol, with the final DNA libraries eluted in 22 μL 10 mM Tris pH 8.0. Library quality and fragment sizes were examined by high sensitivity TapeStation (Agilent) before sequencing.

#### CUT&TAG data analysis pipeline

Basecall data produced by the NextSeq 550 is automatically uploaded to BaseSpace. The FASTQ files were mapped to the human genome (GRCh38) and the spike-in control genome (mm9) using bowtie2 (bwa-0.7.9). The mapped read counts splitting between target species (human) and spike-in control (mm9) were then used for calculating the scaling factors. Next, mapped regions were filtered using SAMtools for the flag of PCR duplication. Filtered BAM files were used for MACS2 calling broadpeak algorithm to identify histone H3K23 and H3K27 acetylation regions respectively, with each BAM file paired with the IgG control from the matching experiment group, (cut off -- mfold 2 50, bandwidth --bw 300, FDR --qvalue 0.05). Peak calling results (gapped peaks) were used for differential peak analysis by applying edgeR differential analysis (fold change >1, FDR-q <0.05) between the control and ALDH1A3 knockdown master list peak reads (*n* = 2), which were compiled by retrieving the filtered BAM reads count from the gapped peak bed coordinates, normalised by sequencing depth, and calibrated using the corresponding scaling factor. R package ChIPseeker^135^ was used for adjacent gene annotation and genomic distribution analysis. Representative peak on gene tracks were visualised using UCSC genome browser following bigwigAverage combining the replicate sample bigwig output from filtered BAM alignment files, which were individually normalised by genome coverage and calibrated using scale factors (bamCoverage --normalizeUsing RPGC --scaleFactor).

##### AlphaFold Multimer modeling of ALD6-ACS2 and ALDH1A3-ACSS2 interaction

Canonical sequences of yeast ALD6 (UniProt: P54115) and ACS2 (UniProt: P52910) and human ALDH1A3 (UniProt: P47895) and ACSS2 (UniProt: Q9NR19) were retrieved.^121^ Disordered N-termini of the human proteins, the first 24 residues of each, were removed to facilitate complex prediction. AlphaFold-Multimer^76^ predictions were performed with LocalColabFold (https://github.com/YoshitakaMo/localcolabfold), running ColabFold version 1.5.2^122^ on a single 350GB NVIDIA A100 GPU. Both yeast and human sequences were run as dimers of ALD6/ALDH1A3 and monomers of ACS2/ACSS2 because of sequence length limitations. We used templates available in the Protein Data Bank^138^ and the “mmseqs2_uniref” option for the –msa-mode flag. For both yeast and human complexes, we generated 3 models with 5 recycles and excluded models that were incompatible with the tetrameric structure of ALDH1A3. Confidence is 45% for yeast and 47% for the human complexes, calculated as 0.2 × pTM +0.8 × ipTM.^76^ The highest ranking models of each complex were further refined with GalaxyRefineComplex,^123^ using default settings of protocol 2. Solvent accessible surface was calculated at residue level with FreeSASA 2.0.3^124^ with the buried surface area defined as the difference in solvent accessible surface area between the monomer and the complex. Homologous residues were determined via sequence alignment with MUSCLE.^139^ Conservation of ACS2/ACSS2 interface with ALD6/ALDH1A3 was determined by calculating the Pearson correlation between the buried surface area values of homologous residues. ATP/CoA binding pocket of ACSS2 was visualised by structural alignment of AlphaFill-optimized^125^ protein-ligand complexes (ATP donor: PDB: 5k8f; CoA donor: PDB: 3gpc) to the AlphaFold-Multimer predicted model. Visualisation of protein structures was performed with UCSF ChimeraX version 1.6.^126^

#### Zebrafish melanoma ALDH activity measurement

Zebrafish melanoma live cells were dissociated from freshly dissected tumor samples as described in Travnickova et al., 2019.^12^ Cell suspensions were sized to 1 million cell count per mL and incubated with AldeRed (ThermoFisher Technologies) following the instruction by the manufacturer. After incubation of 1 h at 28°C, the AldeRed activity was measured using flow cytometry (Fortessa, BD Biosciences). For cell sorting to establish the zebrafish ALDH^High^ and ALDH^Low^ cells, the stained cells were sorted by FACS Aria II (BD Biosciences) and the population with the highest and lowest 5% ALDH activity (ALDH^High^ and ALDH^Low^) were collected, as is described for AldeFluor guided ALDH subpopulation selection.

#### Zebrafish drug pellet treatment

To perform drug treatment on adult zebrafish bearing melanoma, we produced fish bite-size drug pellets as described in our previous publication^81^ and fed single-housed individual fish with fish food agar pellets containing DMSO (Sigma Aldrich), vemurafenib (SelleckChem), and/or Nifuroxazide (Merck Millipore) once per day. Zebrafish under the drug treatment procedure were fed daily in the AM and early PM with artemia, and then fed the drug pellets in the late PM (6–8 p.m.). Zebrafish actively sought for and consumed the drug pellets voluntarily without any handling. Zebrafish under continuous drug treatment were imaged one day pre-treatment and once every week to track tumor size change.

#### Imaging of adult zebrafish tumor and size measurement

Zebrafish were briefly anesthetised (Tricaine in PBS 1:10,000 concentration) for no longer than 10 min per session and fully recovered in fresh system water. Brightfield images were taken for each fish positioned on both sides. Images of fish lesions were captured at the same magnification scale every week using a Nikon COOLPIX5400 camera attached to a brightfield microscope (Nikon SMZ1500). The size of each lesion was quantified by using the manual field selection in Fiji on each tumor image, then compared to the matching pre-treatment lesion to calculate the relative percentage change. Lesions that could be observed from both sides of the fish were measured by combining the area number averaged from both sides.

#### Zebrafish histology and IHC quantification

Zebrafish melanoma samples were collected, fixed, and processed as described in our earlier publications.^12^^,^^19^^,^^140^ The slides of Haematoxylin and Eosin staining were imaged using a Hamamatsu NanoZoomer SlideScanner, and the images were processed using NDP.3 software. Aldh1a3 expression was assessed using Rabbit polyclonal anti-ALDH1A3 primary antibody (1:200, Abcam). Following secondary fluorescent antibody incubation (Donkey anti-mouse conjugated Alexa Fluor 568, Donkey anti-Rabbit conjugated Alexa Fluor 488 or 647, Invitrogen), nuclei were stained with DAPI dye (1:1000, Life Technologies). Stained tissue slides were mounted with antifade mounting medium (Vectashield, 2BScientific) before fluorescent microscope imaging using multimodal Imaging Platform Dragonfly (Andor technologies, Belfast UK). Similar to ICC imaging, images were acquired using a 20X lens equipped with 405, 488, 561, and 640 nm lasers built on a Nikon Eclipse Ti-E inverted microscope body with Perfect focus system (Nikon Instruments, Japan). Data were collected in Spinning Disk 25 μm pinhole mode on the Zyla 4.2 sCMOS camera using a Bin of 1 and no frame averaging using Andor Fusion acquisition software. Standard deviation intensity (STD) projection of a confocal z stack was performed in Fiji to allow intensity quantification and cell subpopulation assessment.

### Quantification and statistical analysis

All statistical methods used in the paper are described in the figure legends and, where indicated, additional details are provided in the method details. Definitions of sample size, measures of center and dispersion, and precision measures are also indicated in figure legends. Statistics were computed using R and GraphPad Prism. When appropriate, corrections for multiple comparisons were implemented as indicated in the figure legends.

## Supplemental information

Document S1. Figures S1–S7, Methods S1, and Data S1

Table S1. ALDH^High^-high vs. ALDH^Low^ Diff genes, related to Figure 1

Table S2. Gene set functional enrichment analysis, related to Figure 1

Table S3. Mass spectrometry, related to Figure 3

Table S4. Diff peak ChIP, related to Figures S3 and S4

Table S5. Gene lists, related to Figures S3 and S4

Table S6. Primers for qPCR, related to STAR Methods oligos

Document S2. Article plus supplemental information
