# ALDH1A3-acetaldehyde metabolism potentiates transcriptional heterogeneity in melanoma

**DOI:** 10.1016/j.celrep.2024.114927

**Published:** 2024-11-14

**Authors:** Yuting Lu, Jana Travnickova, Mihaly Badonyi, Florian Rambow, Andrea Coates, Zaid Khan, Jair Marques, Laura C. Murphy, Pablo Garcia-Martinez, Richard Marais, Pakavarin Louphrasitthiphol, Alex H.Y. Chan, Christopher J. Schofield, Alex von Kriegsheim, Joseph A. Marsh, Valeria Pavet, Owen J. Sansom, Robert S. Illingworth, E. Elizabeth Patton

## Main text

(Cell Reports *43*, 114406; July 23, 2024)

As this article was originally published on July 3, 2024, Figure S6A mislabeled a second set of primers for “*aldh1l2*” as “*aldh1l1*.” The figure has since been updated with the correct labeling online, and a typo for “*aldh9a1b*” has also been corrected. The names for primers probing aldh1l2 have been updated as aldh1l2 (exons 4 and 5), and names of the previous aldh1l1 primers have been corrected to aldh1l2 (exons 5 and 6) so these match with the corrected Figure S6A. This correct figure, along with the original figure, are displayed below for readers’ reference. The authors apologize to their colleagues for this error and appreciate the opportunity to correct the record.

**Figure S6:**
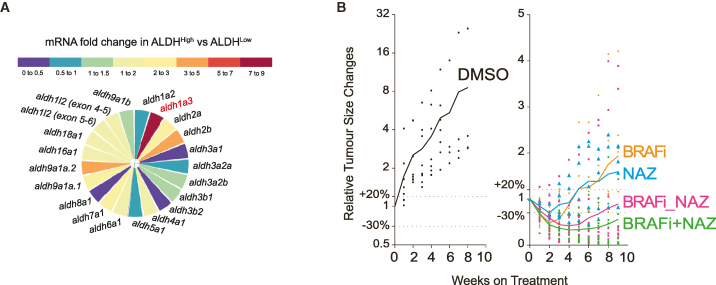
ALDH1A3^High^ subpopulations promote melanoma drug resistance *in vivo*. Related to Figure 7 (corrected)

**Figure S6:**
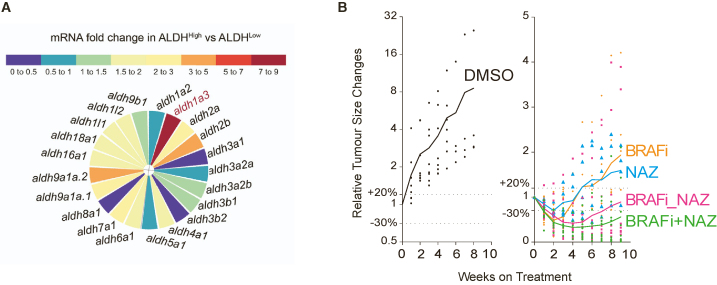
ALDH1A3^High^ subpopulations promote melanoma drug resistance *in vivo*. Related to Figure 7 (original)

