# Supplementary material for: ALDH1A3-acetaldehyde metabolism potentiates transcriptional heterogeneity in melanoma
Source: Cell Rep. 2024 Jul 3;43(7):114406. doi: 10.1016/j.celrep.2024.114406 (PMC11290356; doi:10.1016/j.celrep.2024.114406)
Supplement: Document S1. Figures S1–S7, Methods S1, and Data S1 [file mmc1.pdf]

Cell Reports, Volume *43*

## Supplemental information

# ALDH1A3-acetaldehyde metabolism potentiates transcriptional heterogeneity in melanoma

**Yuting Lu, Jana Travnickova, Mihaly Badonyi, Florian Rambow, Andrea Coates, Zaid Khan, Jair Marques, Laura C. Murphy, Pablo Garcia-Martinez, Richard Marais, Pakavarin Louphrasitthiphol, Alex H.Y. Chan, Christopher J. Schofield, Alex von Kriegsheim, Joseph A. Marsh, Valeria Pavet, Owen J. Sansom, Robert S. Illingworth, and E. Elizabeth Patton**

**Supplementary Table Titles**

**Table S1. ALDH High vs Low Diff genes related to Figure 1**

**Table S2. GSEA related to Figure 1**

**Table S3. Mass spec related to Figure 3**

**Table S4. Diff peak ChIP related to Figure S3-S4**

**Table S5. Genelists related to Figure S3 and Figure 4**

**Table S6. Primers for qPCR Related to STAR Methods Oligos**

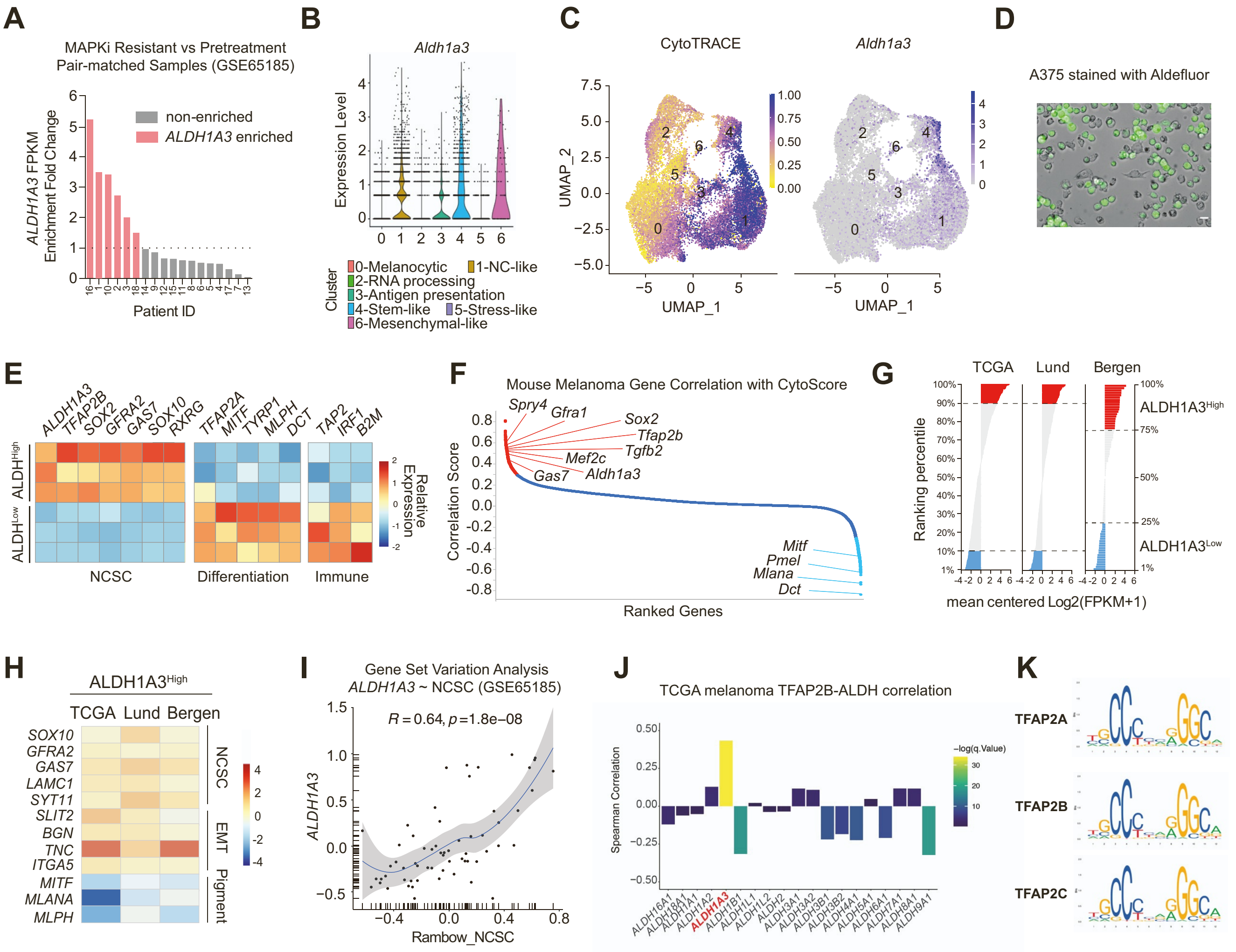


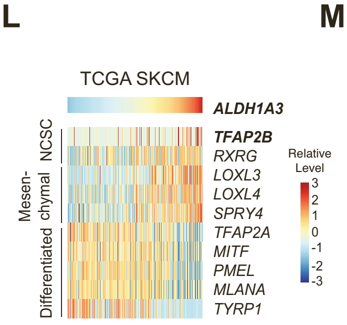


**M**

| Sample ID | Age/sex | Mutation status | Pre-operative treatment | Site of resection |
|---|---|---|---|---|
| Melanoma_53 | 77/F | Wild-type | None | Subcutaneous back lesion |
| Melanoma_59 | 80/M | Wild-type | None | Femoral lymph node |
| Melanoma_60 | 69/M | BRAF V600K | Trametinib, ipilimumab | Spleen |
| Melanoma_65 | 65/M | BRAF V600E | None | Paraspinal intramuscular |
| Melanoma_67 | 58/M | BRAF V600E | None | Axillary lymph node |
| Melanoma_71 | 79/M | NRAS Q61L | None | Transverse colon |
| Melanoma_78 | 73/M | NRAS Q61L | WDVAX, ipilimumab + nivolumab | Small bowel |
| Melanoma_79 | 74/M | Wild-type | None | Axillary lymph node |
| Melanoma_80 | 86/F | NRAS Q61L | None | Axillary lymph node |
| Melanoma_81 | 43/F | BRAF V600E | None | Axillary lymph node |
| Melanoma_82 | 81/M | Wild-type | None | Axillary lymph node |
| Melanoma_84 | 67/M | Wild-type | None | Acral primary |
| Melanoma_88 | 54/F | NRAS Q61L | Tremelimumab + MEDI3617 | Cutanoues met |
| Melanoma_89 | 67/M | n/a | None | Axillary lymph node |
| Melanoma_94 | 54/F | Wild-type | IFN, ipilimumab + nivolumab | Iliac lymph node |

**Lu et al., Supplementary Figure 1**

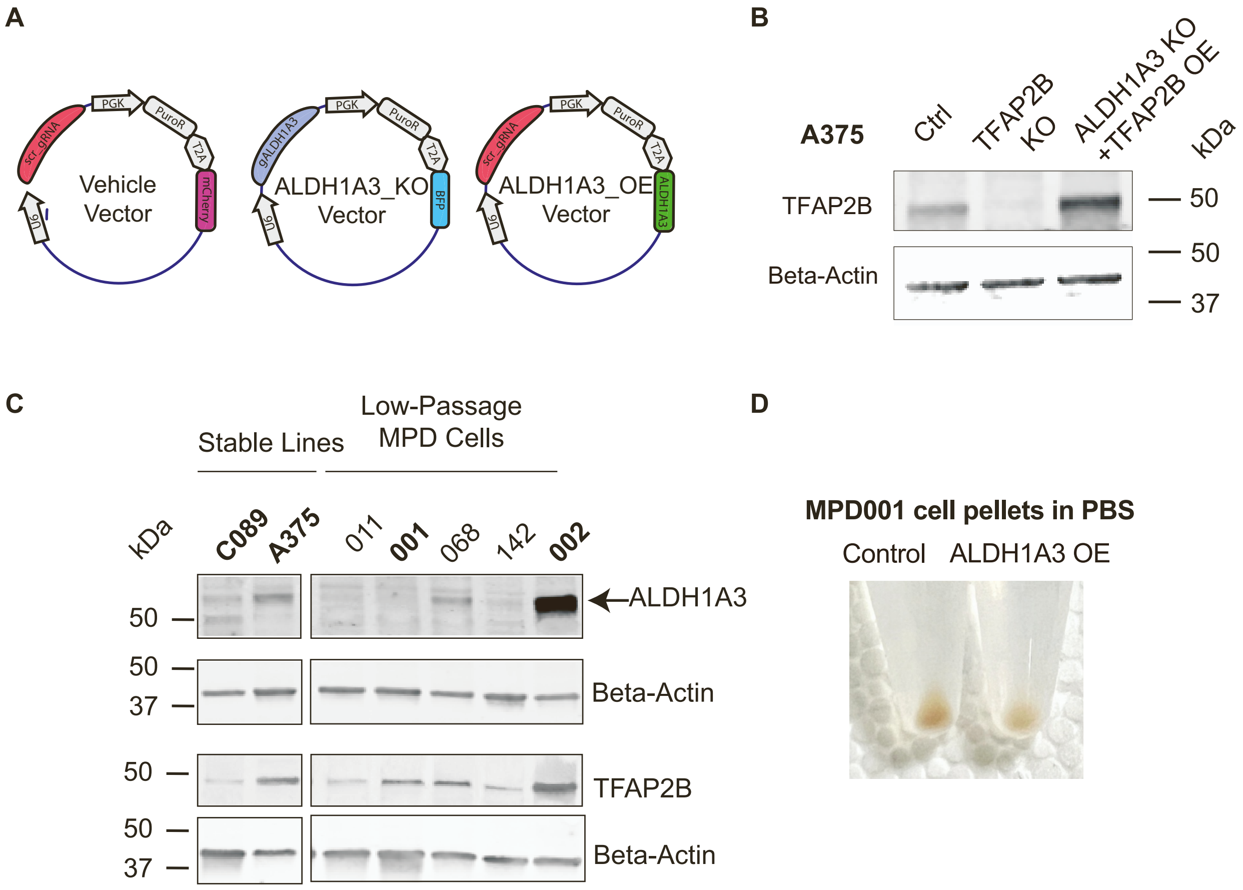


Lu et al., Supplementary Figure 2

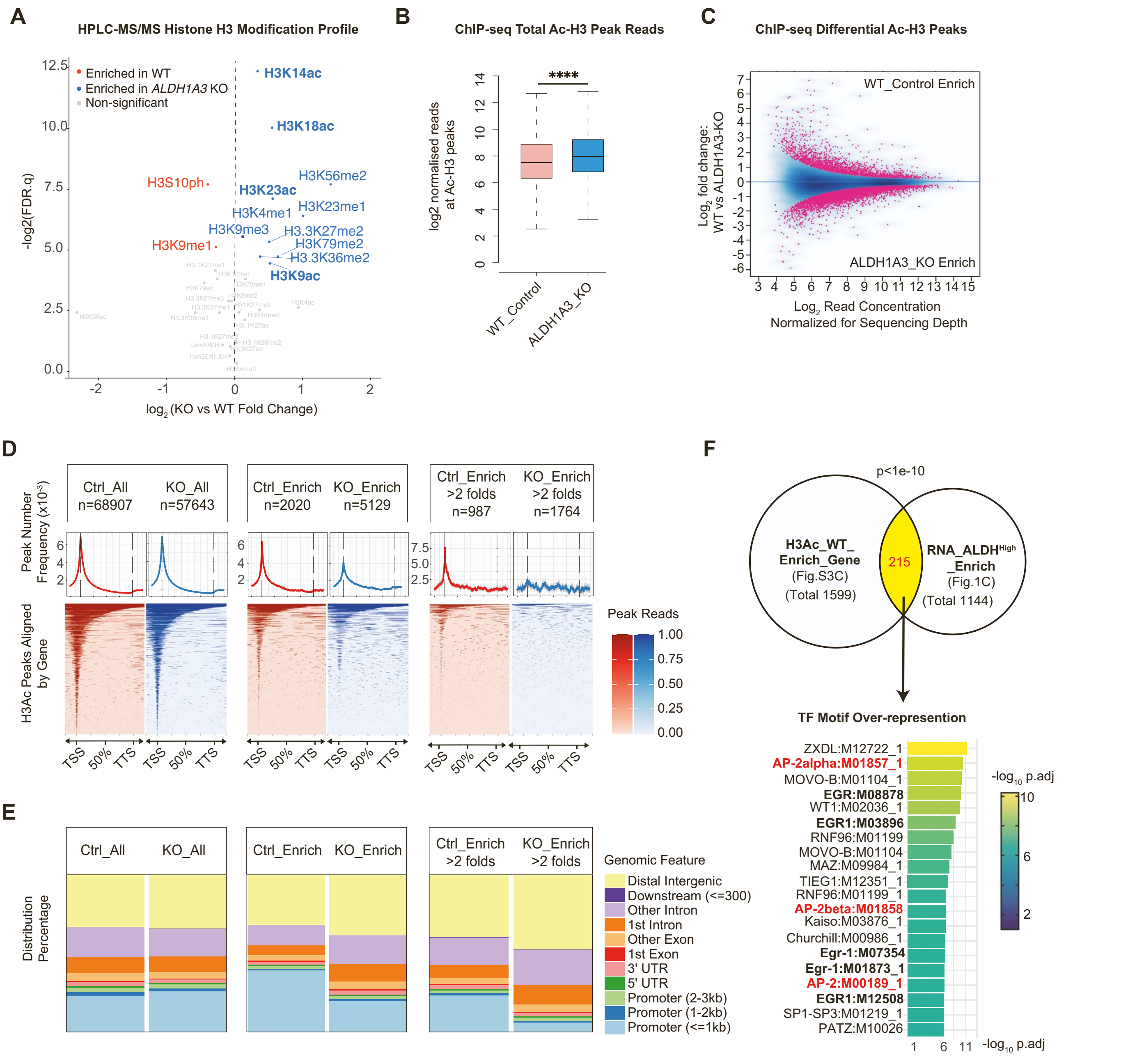


**Lu et al., Supplementary Figure 3**

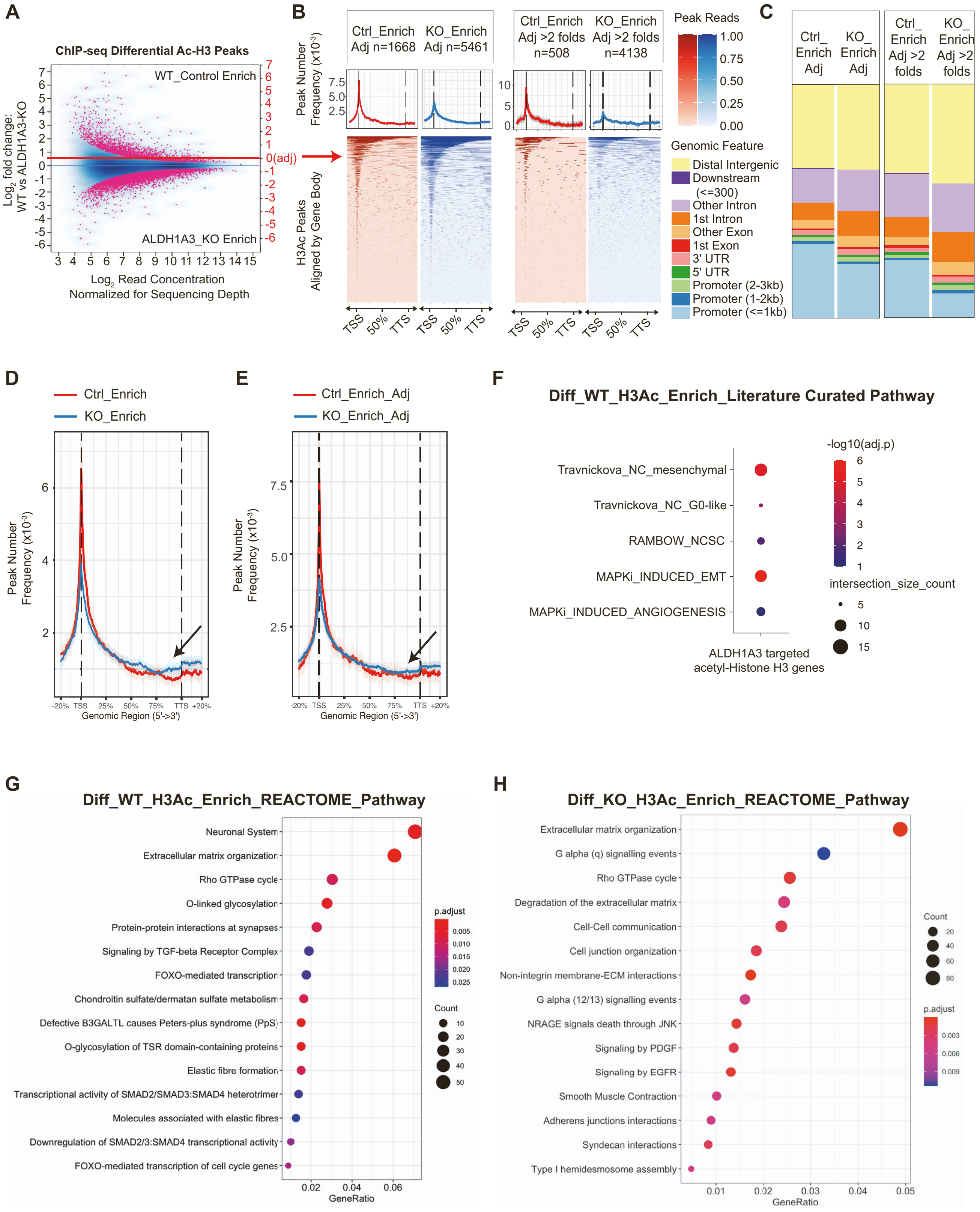


**Lu et al., Supplementary Figure 4**

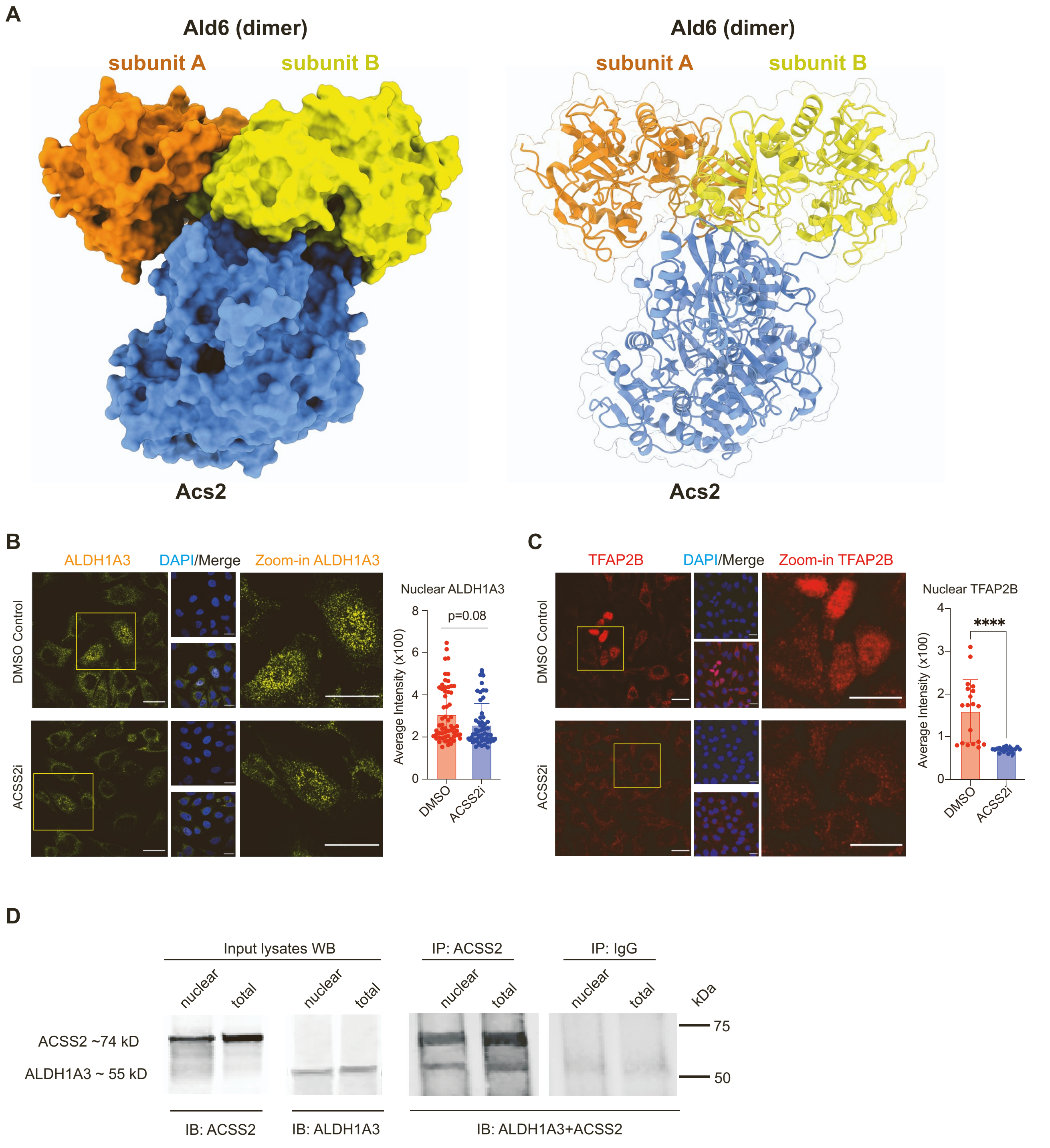


Lu et al., Supplementary Figure 5

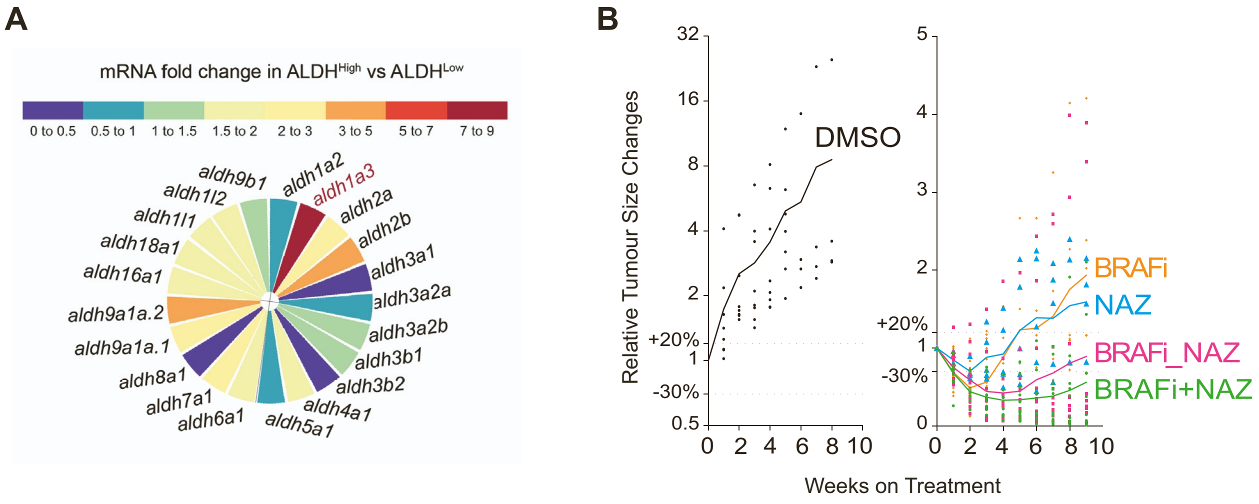


Lu et al., Supplementary Figure 6

**A**

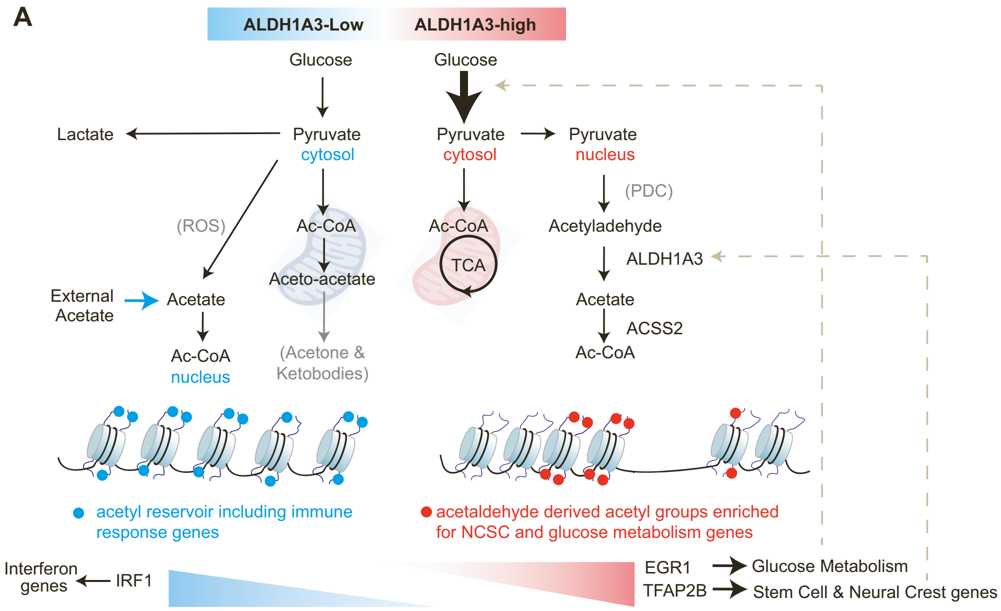


**B**

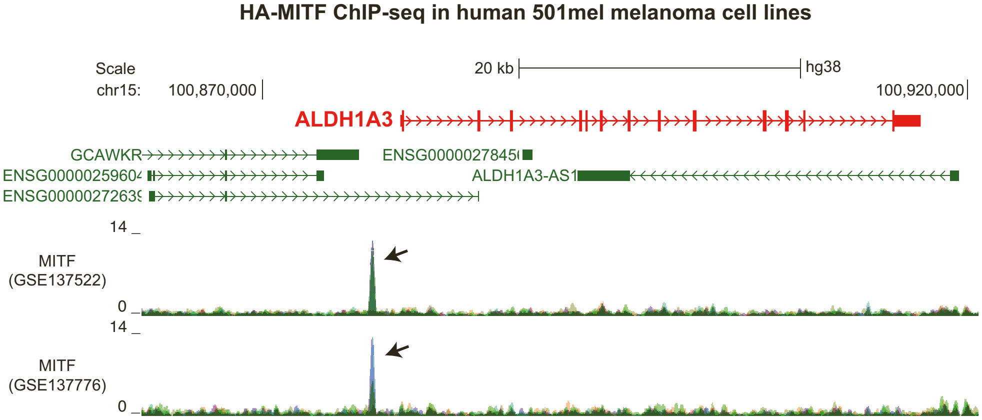


**Lu et al., Supplementary Figure 7**

**Supplementary Figure Legends**

**Supplementary Figure 1: ALDH1A3$^{High}$ melanomas are enriched for NCSC and glucose metabolic states. Related to Figure 1**

**A.** Pair-matched patient samples (GSE65185)[1] ranked by *ALDH1A3* fold enrichment comparing post MAPKi resistance to pre-treatment biopsies. The average of *ALDH1A3* was used for comparison when multiple MAPKi-resistant tumour biopsies were taken from the same patient.

**B.** Violin plot depicts *Aldh1a3* RNA expression in different murine melanoma cell states, including NC-like (cluster 1) and stem-like states (cluster 4)[2].

**C.** UMAP projection of murine melanoma cells colored by CytoTRACE score (gene expression diversity score, less differentiated = score close to 1 and more differentiated = score close to 0) and Aldh1a3 expression. Cluster numbers that relate to **B** are indicated.

**D.** Microscope image overlaying brightfield and GFP channel shows the heterogeneous ALDH activity among A375 melanoma cells as measured by the Aldefluor assay on (adhered) live cells. Scale bar = 20 μm.

**E.** Heatmap showing RT-qPCR results validating differentially expressed genes identified from RNA-seq on 3 bio-replicants of independently sorted A375 ALDH$^{High}$ and ALDH$^{Low}$ cells. Relative expression of each gene was averaged across 3 technical replicants and normalised to beta-actin loading control before heatmap plotting. For heatmap visualisation, each row (each gene) was scaled for the colour index filling.

**F.** Murine melanoma genes from **B, C** ranked by decreasing correlation coefficient with CytoTRACE score (genes conferring a less differentiated phenotype close to 1 and genes conferring a differentiated phenotype close to -1).

**G.** Patient samples ranked by *ALDH1A3* expression, with the top and bottom 10% ranking samples of TCGA and Lund datasets defined as ALDH1A3$^{High}$ and ALDH1A3$^{Low}$ group respectively, while the top and bottom 25% ranking samples of Bergen datasets were defined as ALDH1A3$^{High}$ and ALDH1A3$^{Low}$ groups due to fewer patient numbers in the Bergen cohort.

**H.** Heatmap showing gene expression fold enrichment levels comparing the ALDH1A3$^{High}$ to ALDH1A3$^{Low}$ patient groups. Colorimetric values normalized by column (each cohort).

**I.** Scatter plot showing the correlation of *ALDH1A3* and NCSC gene set expression (measured by gene set variation analysis) over different human patient melanoma samples (GSE65185). Spearman's rank correlation score (*R*) and probability (p) value.

**J.** Bar plot showing spearman correlation value of each *ALDH* isoform with *TFAP2B* in TCGA dataset. Colour filling scaled by FDR.q.value. Only *ALDH1A3* shows significant positive correlation with TFAP2B.

**K.** DNA Binding motif sequences of TFAP2 family members from JASPAR database.

**L.** *ALDH1A3* gene network heatmap. Heatmap of TCGA melanoma samples with RNA expression of *TFAP2B*, *RXRG* and mesenchymal genes, as well as *MITF*, *TFAP2A*, and their target genes ranked by *ALDH1A3* level. Colour index filling scaled by row (each gene).

**M.** Mutation and clinical background patient sample table from Tirosh et al., 2016[3]. The cluster of *ALDH1A3-TFAP2B* consists of cells from highlighted patient entries.

**Supplementary Figure 2: Perturbation of ALDH1A3 and TFAP2B in melanoma. Related to Figure 2**

**A.** Schematics of plasmids used to generate wildtype (WT), *ALDH1A3* knockout (KO), and *ALDH1A3* overexpression (OE) cell lines.

**B.** Western blot of TFAP2B levels in *TFAP2B* knockout cells and *ALDH1A3* knockout cells overexpressing *TFAP2B.* Beta-Actin is presented as a loading control.

**C.** Western blot of ALDH1A3 and TFAP2B levels across established stable melanoma cell lines used for this work (C089 and A375) as well as a panel of MPD low passage cells.

**D.** *ALDH1A3* overexpression leads to less pigmentation. Cell pellets showing brown melanin in control and ALDH1A3 overexpressing MPD001 cells.

**Supplementary Figure 3: Histone acetylation distribution is dependent on ALDH1A3. Related to Figure 3-4**

**A.** Volcano plot of histone H3 peptide modification quantification measured by bottom-up histone HPLC-MS/MS. (n=5, FDR q values by two-way ANOVA corrected with Sidak's multiple comparison method.) Acetyl-histone H3 lysine residuals with significant upregulation in *ALDH1A3 KO* group comparing to the control are highlighted in **Bold**.

**B.** Collective acetyl-histone H3 reads abundance at peak regions measured by acetyl-histone H3 ChIP-seq. (Wilcoxon signed-rank test. ****P<0.0001).

**C.** Volcano plot of differentially acetylated histone H3 peaks in WT control and ALDH1A3 knockout (highlighted by magenta, fold change >1, FDR.q < 0.05).

**D. E. (D)** Heatmap of ChIP-seq histone pan-acetyl-histone H3 peaks aligned by gene body extended by 20% upstream transcription starting site (TSS) and downstream transcription termination site (TTS) (bottom panels), with average profile showing read count frequency distribution (upper panels) and **(E)** genomic annotations by bar plot (top panels) among A375 control (Ctrl_All), ALDH1A3 knockout (KO_All), significantly enriched peaks in A375 control (Ctrl_Enrich, Ctrl_Enrich>2 folds), and significantly enriched peaks in ALDH1A3 knockout (KO_Enrich, KO_Enrich>2 folds).

**F.** (Upper panel) Venn diagram of overlapping genes between upregulated genes by RNA-Seq in sorted $ALDH^{High}$ cells **(Figure 1C)**, and genes enriched in control cells versus *ALDH1A3* knockout cells by histone H3 acetylation in **B**. P values by Fisher's exact test. (lower panel) Transcription factor binding motif over-representation analysis of the Venn diagram overlapping genes ranked by enrichment score calculated from g:Profiler (version e109_eg56_p17_1d3191d) with g:SCS multiple testing correction method applying statistical significance threshold of 0.05 [4].

**Supplementary Figure 4: Stringent pan-histone H3 acetylation ChIP-seq analysis. Related to Figure 4 and Figure S3**

**A.** Schematics of adjusted (right Y axis, highlighted in red) differential peak analysis on acetylated histone H3 peaks in WT control compared to *ALDH1A3* knockout cells. Here, we have manually increased log base 2 peak score by +0.5 in *ALDH1A3* KO samples to account for the potential biased loss of peak score during normalisation.

**B.** Heatmap of adjusted differentially acetylated histone H3 peaks aligned by the gene body and extended by 20% upstream transcription starting site (TSS) and downstream of the transcription termination site (TTS) (bottom panels). Read count frequency distribution is shown in an average profile (upper panels).

**C.** Genomic annotations by bar plot among adjusted enriched peaks in A375 control (Ctrl_Enrich_Adj, Ctrl_Enrich_Adj>2 folds), and in *ALDH1A3* knockout cells (KO_Enrich_Adj, KO_Enrich_Adj>2 folds).

**D-E.** Overlay of the differentially enriched acetyl-histone H3 peaks aligned by gene body from **Figure 4C** and **(E)** from **Figure S4B.** Both showed reduced proportion of TSS-centring peaks in *ALDH1A3* KO cells, coupled with increased proportion of peaks towards the TTS and extended gene downstream regions as highlighted by arrows.

**F-H.** Significant over-representation gene terms with literature curated gene lists **(F).** Reactome gene pathway datasets enriched in control **(G)** and *ALDH1A3* knockout **(H)** cells. FDR <0.05, Benjamini-Hochberg test.

**Supplementary Figure 5: ALDH1A3 forms a predicted complex with ACSS2. Related to Figure 5**

**A.** AlphaFold multimer modelling of yeast Ald6 and Acs2 proteins visualised in surface model (left) and ribbon model (right).

**B-C**. ICC staining by fluorescence antibody for ALDH1A3 (yellow, in **B**), and TFAP2B (red, in **C**) in sorted A375 $ALDH^{High}$ and $ALDH^{Low}$ cells. DAPI (blue). Scale bar = 20 μm. Fluorescence signal intensity quantification of nuclear **(B)** ALDH1A3 and **(C)** TFAP2B in ICC images. n = 60 single cells for ALDH1A3 and n=19 single cells for TFAP2B quantification (represented as individual dots), mean±s.d., unpaired non-parametric Kolmogorov-Smirnov test. ****P<0.0001.

**D.** Western blot of nuclear or total A375 cell lysates probing either ACSS2 or ALDH1A3 levels of the input materials used for co-immunoprecipitation of ACSS2 (IP: ACSS2 and IP: IgG). The co-immunoprecipitated materials were examined by immunoblotting ALDH1A3 as well as ACSS2. Immunoprecipitation of ACSS2 successfully captured ALDH1A3, and this was not detected in the IgG control. For each condition (isolated nuclei versus whole cell department), the input, IP: ACSS2, and IP: IgG materials were divided from the same vial of harvested A375 cells.

**Supplementary Figure 6: ALDH1A3$^{High}$ subpopulations promote melanoma drug resistance *in vivo*. Related to Figure 7**

**A.** RT-qPCR quantification shown by a heat rose map of *aldh1a3* expression enriched with the highest folds in zebrafish ALDH$^{High}$ versus ALDH$^{Low}$ melanoma cells. (n=3 bio-replicates each with 3 technical replicates, Multiple paired t-test corrected with Holm-Sidak's method).

**B.** Spaghetti summary plot related to **Figure 7H**, showing tumour volume changes during drug trial design as listed in **Figure 7G**. DMSO: melanomas n=8, fish N=5; BRAFi: melanomas n=5, fish N=4; NAZ: melanomas n=6, fish N=4. BRAF_NAZ: melanomas n=12, fish N=5; BRAFi + NAZ: melanomas n=10, fish N=4. Each dot on the plot represents one melanoma lesion. Drug pellet treatment is colour coded as shown in **G**: DMSO (Black): daily DMSO control treatment. BRAFi (Orange): 200 mg/kg/day vemurafenib treatment. NAZ (Blue): daily 150 mg/kg/day Nifuroxazide treatment. BRAFi_NAZ (Magenta): 3-week treatment of 200 mg/kg/day vemurafenib followed by daily 150 mg/kg/day Nifuroxazide treatment. BRAFi+NAZ (Green): 3-week treatment of 200 mg/kg/day vemurafenib followed by the combination of 200 mg/kg/day vemurafenib and 150 mg/kg/day Nifuroxazide treatment.

**Supplementary Figure 7: Model of ALDH1A3 regulating melanoma heterogeneity**

**A.** Schematic model of ALDH1A3 orchestrating the metabolic, epigenetic, and transcriptional cell states of melanoma.

**B.** UCSC genome browser snapshot of MITF ChIP-seq multiWig tracks at the ALDH1A3 locus from two experiments (Top, GSE137522; Bottom, GSE137776). Each track is an overlay of an input control and 2 independent HA-MITF ChIP-seq experiments in the human 501mel melanoma cell line engineered to inducibly express HA-tagged MITF. Inputs are shown in Green Mist colour RGB (200,200,150).

## Supplementary Methods 1: Acetyl-histone H3 ChIP-seq analysis in WT and *ALDH1A3* KO melanoma cells. Related to Figure S3 and S4.

To determine how ALDH1A3-dependent histone H3 acetylation is deposited on chromatin, we first performed quantitative acetyl-histone H3 chromatin immunoprecipitation (ChIP)-seq using an antibody against pan-histone H3 acetylated sites (K9 + K14 + K18 + K23 + K27). We found that the average acetyl-histone H3 signal increased by ~1.4 fold across H3-ac peak regions in *ALDH1A3* KO cells **(Figure S3B;** consistent with our western blotting and mass spectrometry analysis, **Figure 3K, L; Figure S3A),** and identified 5129 acetyl-histone H3 peaks enriched in *ALDH1A3* KO cells, versus 2020 peaks enriched in *ALDH1A3* control cells (fold change >1, FDRq < 0.05) **(Figure S3C, D).** Notably, in control cells, the enriched acetyl-histone H3 peaks were clustering around transcription start sites (TSSs), especially within 1kb of promoters, whereas in *ALDH1A3* KO cells, the enriched acetyl-histone H3 peaks were broadly dispersed throughout the genome, and particularly spreading into the distal intergenic region and intronic regions **(Figure S3D, E)**. This distribution effect is even more prominent when comparing the top fold-change enriched acetyl-histone H3 peaks (>2 fold) between control and *ALDH1A3* KO cells **(Figure S3D, E)**. To test the robustness of our differential acetyl-histone H3 analysis, we adjusted the average peak score of *ALDH1A3* KO samples by manually increasing the log base 2 value by +0.5, ~ log2(1.4) **(Figure S4A)**, which is the fold change determined by western blot and mass spec **(Figure 3K, L; Figure S3A).** With the adjusted peak score (a more stringent analysis), enriched acetyl-histone H3 remained distributed at distal intergenic and intronic regions in the *ALDH1A3* KO, in contrast with the enriched acetyl-histone H3 in promoter areas in control cells **(Figure S4B-E).**

To determine the mechanistic basis of information flow from acetyl-histone H3 to transcription in cells with high ALDH1A3, we compared genes with promoters marked by high acetyl-histone H3 (total 1499) with the RNA-seq from **Figure 1C.** We found that ~19% of genes with enriched

expression in ALDH1A3$^{High}$ (total 1144) have ALDH1A3-driven acetyl-histone H3 in their promoters (215, $p<1e-10$) **(Figure S3F)**.

In addition, and consistent with our prior observations **(Figure 1, Figure 3),** pathway enrichment analysis revealed that neural crest and stem cell state marker genes, neuronal signalling, TGF-beta signalling, and O-glycosylation pathways were over-represented in ALDH1A3$^{High}$ specific acetyl-histone H3 states **(Figure S4F, G).** Pathways enriched in acetyl-histone H3 sites in ALDH1A3 KO cells also included cell-cell junction terms **(Figure S4H)**, possibly reflecting the difference in cell morphology between the rounded ALDH$^{High}$ cells and the flat, elongated ALDH$^{Low}$ cells **(Figure 1B).** Together, these observations support that the high-glucose flux and NCSC transcriptional states promoted by high ALDH activity arise from selective histone H3 acetylation.

**Supplementary Data 1: The synthesis and NMR spectrum for AC-148. Related to Figure 6.**

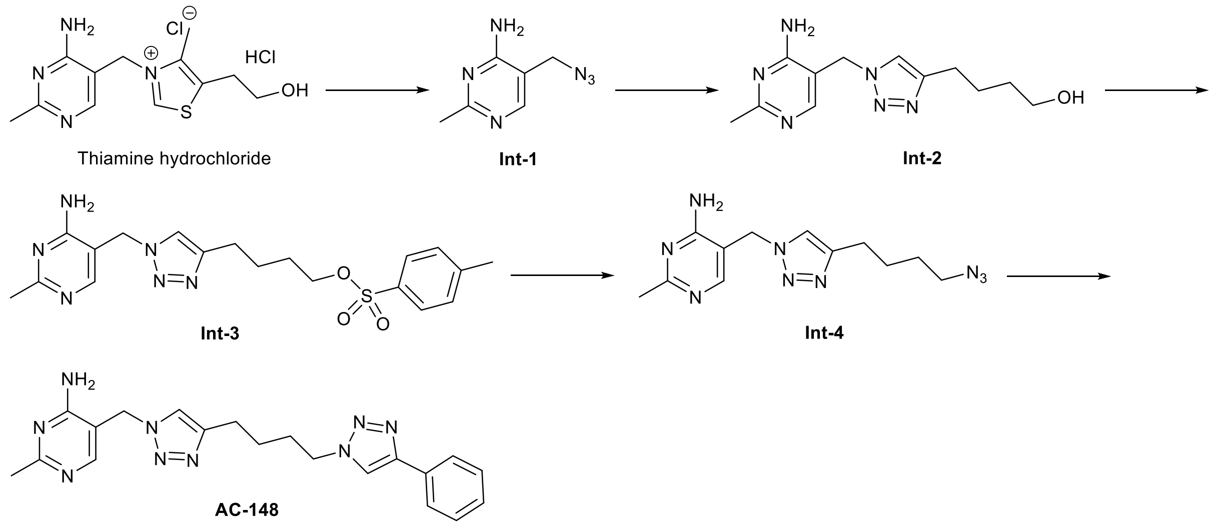


*5-(Azidomethyl)-2-methylpyrimidin-4-amine* **Int-1**

To a stirred solution of thiamine hydrochloride (10.0 g, 29.7 mmol) and $NaN_3$ (5.0 g, 76.9 mmol) in water (90 mL, 0.33 M) was added $Na_2SO_3$ (0.4 g, 3.2 mmol). The resultant mixture was stirred at 70 °C for 18 hours, then acidified with citric acid monohydrate (10.5 g) to pH 4-5, washed with DCM (200 mL), and basified with $K_2CO_3$ to pH 8-10. Upon product precipitation, the suspension was filtered under reduced pressure. The residue was rinsed with cold water and dried under reduced pressure to yield **Int-1** as a white solid (3.4 g, 70%). **$^1$H NMR** (400 MHz, $CD_3OD$) δ 8.02 (s, 1H), 4.32 (s, 2H), 2.43 (s, 3H). Analytical data are consistent with those reported [5].

*4-{1-[(4-Amino-2-methylpyrimidin-5-yl)methyl]-1H-1,2,3-triazol-4-yl}butan-1-ol* **Int-2**

To a stirred solution of **Int-1** (1322 mg, 8.0 mmol) and 5-hexyn-1-ol (1020 mg, 10.4 mmol) in *t*-BuOH and water (24 + 8 mL, 0.25 M) was added $CuSO_4{\cdot}5H_2O$ (60 mg, 0.24 mmol) and sodium ascorbate (475 mg, 2.4 mmol). The resultant mixture was stirred at 40 °C for 40 hours, concentrated under reduced pressure, diluted with $CHCl_3$/*i*-PrOH (3:1, 50 mL), washed with 0.1 M $K_2CO_3$ (50 mL), dried over anhydrous $Na_2SO_4$, filtered, and evaporated under reduced pressure. The residue was purified by silica flash chromatography (10% MeOH in DCM) to yield **Int-2** as a white solid (1366 mg, 65%). **$^1$H NMR** (400 MHz, $CD_3OD$) δ 8.03 (s, 1H), 7.80 (s, 1H), 5.47 (s, 2H), 3.56 (t, 2H, $J$ = 6.5 Hz), 2.71 (t, 2H, $J$ = 7.7 Hz), 2.42 (s, 3H), 1.73 (m, 2H), 1.57 (m, 2H). Analytical data are consistent with those reported [5].

*4-{1-[(4-Amino-2-methylpyrimidin-5-yl)methyl]-1H-1,2,3-triazol-4-yl}butyl 4-methylbenzene-1-sulfonate* **Int-3**

To a stirred solution of **Int 2** (1337 mg, 5.1 mmol) in dry pyridine (25.5 mL, 0.2 M) under nitrogen at 0 °C was added *p*-TsCl (4850 mg, 25.5 mmol) in three portions. The resultant mixture was stirred at 25 °C for 4 hours, quenched with cold 1 M HCl (20 mL), diluted with water (10 mL), neutralised with $NaHCO_3$ to pH 7, and extracted with DCM (150 mL). The organic phase was washed with sat. aq. $Cu_2SO_4$ (100 mL), dried over anhydrous $Na_2SO_4$, filtered, and evaporated under reduced pressure. The residue was purified by silica flash chromatography (10% MeOH in DCM) to yield **Int-3** as a pale-yellow semi-solid (1063 mg, 50%). **$^1$H NMR** (400 MHz, $CD_3OD$) δ

8.04 (s, 1H), 7.78 (d, 2H, *J* = 7.8 Hz), 7.75 (s, 1H), 7.43 (d, 2H, *J* = 7.8 Hz), 5.46 (s, 2H), 4.04 (t, 2H, *J* = 5.5 Hz), 2.63 (t, 2H, *J* = 6.5 Hz), 2.45 (s, 3H), 2.42 (s, 3H), 1.65 (m, 4H). Analytical data are consistent with those reported [5].

*5-{[4-(4-Azidobutyl)-1H-1,2,3-triazol-1-yl]methyl}-2-methylpyrimidin-4-amine* **Int-4**

To a stirred solution of **Int 3** (1041 mg, 2.5 mmol) in dry DMF (2.5 mL, 1 M) under nitrogen was added $NaN_3$ (325 mg, 5.0 mmol). The resultant mixture was stirred at 25 °C for 40 hours, quenched with 0.1 M $K_2CO_3$ (50 mL), and extracted with $CHCl_3$/*i*-PrOH (3:1, 50 mL). The organic phase was dried over anhydrous $Na_2SO_4$, filtered, and evaporated under reduced pressure. The residue was purified by silica flash chromatography (10% MeOH in DCM) to yield **Int-4** as a white foam (445 mg, 62%). **$^1$H NMR** (400 MHz, $CD_3OD$) δ 8.04 (s, 1H), 7.81 (s, 1H), 5.47 (s, 2H), 3.33 (t, 2H, *J* = 6.7 Hz), 2.74 (t, 2H, *J* = 7.6 Hz), 2.42 (s, 3H), 1.74 (m, 2H), 1.62 (m, 2H). Analytical data are consistent with those reported [5].

*2-Methyl-5-({4-[4-(4-phenyl-1H-1,2,3-triazol-1-yl)butyl]-1H-1,2,3-triazol-1-yl}methyl)pyrimidin-4-amine* **AC-148**

To a stirred solution of Int-4 (86 mg, 0.3 mmol) and phenylacetylene (31 mg, 0.3 mmol) in *t*-BuOH and water (0.9 + 0.3 mL, 0.25 M) was added $CuSO_4 \cdot 5H_2O$ (2.5 mg, 0.01 mmol) and sodium ascorbate (20 mg, 0.1 mmol). The resultant mixture was stirred at 40 °C for 72 hours, concentrated under reduced pressure, diluted with EtOAc (50 mL), washed with 0.1 M $K_2CO_3$ (50 mL), dried over anhydrous $Na_2SO_4$, filtered, and evaporated under reduced pressure. The residue was purified by silica flash chromatography (10% MeOH in DCM) to yield **AC-148** as a white solid (41 mg, 35%). **$^1$H NMR** (400 MHz, $CD_3SOCD_3$) δ 8.57 (s, 1H), 7.94 (s, 1H), 7.82-7.86 (m, 3H), 7.41-7.47 (m, 2H), 7.30-7.35 (m, 1H), 6.87 (br, 2H, $NH_2$), 5.37 (s, 2H), 4.42 (t, 2H, *J* = 6.9 Hz), 2.65 (t, 2H, *J* = 7.8 Hz), 2.30 (s, 3H), 1.86-1.96 (m, 2H), 1.54-1.63 (m, 2H). **HRMS** (ESI) m/z: [M+H$^+$] calculated for C20H24N9: 390.2149; found: 390.2146. Analytical data are consistent with those reported [5].

$^{1}$H NMR of **AC-148** in $CD_3SOCD_3$:

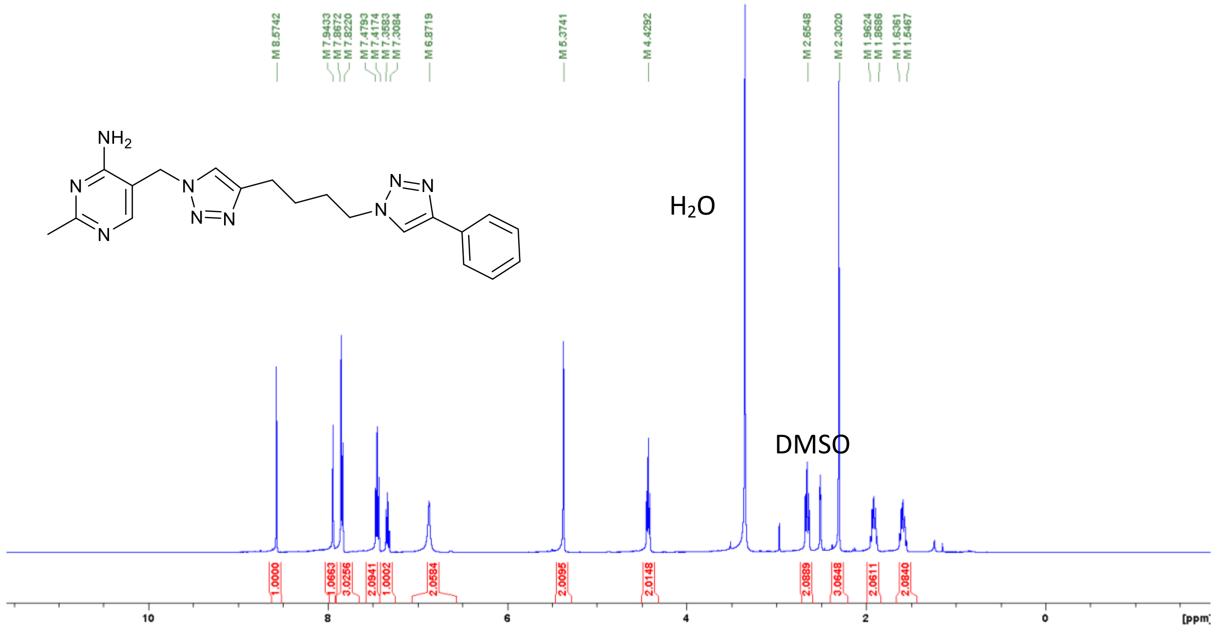



1. Hugo, W., Shi, H., Sun, L., Piva, M., Song, C., Kong, X., Moriceau, G., Hong, A., Dahlman, K.B., Johnson, D.B., et al. (2015). Non-genomic and Immune Evolution of Melanoma Acquiring MAPKi Resistance. Cell *162*, 1271-1285. 10.1016/j.cell.2015.07.061.

2. Karras, P., Bordeu, I., Pozniak, J., Nowosad, A., Pazzi, C., Van Raemdonck, N., Landeloos, E., Van Herck, Y., Pedri, D., Bervoets, G., et al. (2022). A cellular hierarchy in melanoma uncouples growth and metastasis. Nature *610*, 190-198. 10.1038/s41586-022-05242-7.

3. Tirosh, I., Izar, B., Prakadan, S.M., Wadsworth, M.H., 2nd, Treacy, D., Trombetta, J.J., Rotem, A., Rodman, C., Lian, C., Murphy, G., et al. (2016). Dissecting the multicellular ecosystem of metastatic melanoma by single-cell RNA-seq. Science *352*, 189-196. 10.1126/science.aad0501.

4. Raudvere, U., Kolberg, L., Kuzmin, I., Arak, T., Adler, P., Peterson, H., and Vilo, J. (2019). g:Profiler: a web server for functional enrichment analysis and conversions of gene lists (2019 update). Nucleic Acids Res *47*, W191-W198. 10.1093/nar/gkz369.

5. Chan, A.H.Y., Ho, T.C.S., Fathoni, I., Pope, R., Saliba, K.J., and Leeper, F.J. (2023). Inhibition of Thiamine Diphosphate-Dependent Enzymes by Triazole-Based Thiamine Analogues. ACS Med Chem Lett *14*, 621-628. 10.1021/acsmedchemlett.3c00047.
